# Characterization of ASR gene and its role in drought tolerance in chickpea (*Cicer arietinum* L.)

**DOI:** 10.1371/journal.pone.0234550

**Published:** 2020-07-14

**Authors:** Supriya Sachdeva, C. Bharadwaj, Rajesh Kumar Singh, P. K. Jain, B. S. Patil, Manish Roorkiwal, Rajeev Varshney

**Affiliations:** 1 Division of Genetics, Indian Agricultural Research Institute (ICAR), Pusa, New Delhi, India; 2 National Institute of Plant Biotechnology (ICAR), Pusa, New Delhi, India; 3 International Crops Research Institute for Semi Arid Crops (ICRISAT), Patancheru, Telangana, India; Louisiana State University, UNITED STATES

## Abstract

Chickpea has a profound nutritional and economic value in vegetarian society. Continuous decline in chickpea productivity is attributed to insufficient genetic variability and different environmental stresses. Chickpea like several other legumes is highly susceptible to terminal drought stress. Multiple genes control drought tolerance and ASR gene plays a key role in regulating different plant stresses. The present study describes the molecular characterization and functional role of Abscissic acid and stress ripening (ASR) gene from chickpea (*Cicer arietinum*) and the gene sequence identified was submitted to NCBI Genbank (MK937569). Molecular analysis using MUSCLE software proved that the ASR nucleotide sequences in different legumes show variations at various positions though ASR genes are conserved in chickpea with only few variations. Sequence similarity of ASR gene to chickpea putative ABA/WDS induced protein mRNA clearly indicated its potential involvement in drought tolerance. Physiological screening and qRT-PCR results demonstrated increased ASR gene expression under drought stress possibly enabled genotypes to perform better under stress. Conserved domain search, protein structure analysis, prediction and validation, network analysis using Phyre2, Swiss-PDB viewer, ProSA and STRING analysis established the role of hypothetical ASR protein NP_001351739.1 in mediating drought responses. NP_001351739.1 might have enhanced the ASR gene activity as a transcription factor regulating drought stress tolerance in chickpea. This study could be useful in identification of new ASR genes that play a major role in drought tolerance and also develop functional markers for chickpea improvement.

## Introduction

Chickpea (*Cicer arietinum* L.), one of the earliest food legume crop with a diploid chromosome number of 16 is cultivated in the tropics all over the world [1] and belongs to the family Fabaceae [2]. India being the largest producer of chickpea produces 68% of the total world production and about 9.21Mha area is under chickpea cultivation producing 8.88Mt [3]. Chickpea, characterized by different desi and kabuli cultivars has a profound nutritional and economic value [4]. A considerable decrease in chickpea productivity has been observed in the last thirty years due to change from lower to higher temperature regions of cultivation in South-East Asia and East Africa [5]. Presently, the world average productivity is about 995 kg/ha which is very low [3] and has stagnated in recent years due to vulnerability of chickpea crop to various abiotic (drought, terminal heat, high salt, cold stress), and biotic (*Ascochyta* blight, *Fusarium* wilt, *Helicoverpa*) stresses [6]. Average losses upto 60% have been reported due to abiotic stresses globally in chickpea [7]. Drought drastically affects the plant growth processes and reduces plant yield [8]. Development of chickpea varieties tolerant to drought has been very slow due to its narrow base and limited genomic resources [9] necessitate improving its genetic potential [10]. Plants combat these stresses through a series of physiological mechanisms controlled by several stress related genes which in-turn is regulated by specific transcription factors [11–13].

Drought is a genetically complex trait [14]. Among various transcription factors, abscissic acid is involved in signaling drought stress in general and their levels have a direct effect on different parts of the plant or plant as a whole [15]. Drought tolerance may be governed by some pathways that are ABA dependent [16] or pathways that are independent of ABA [17]. Particularly, transcription factors of the Asr (abscissic acid, stress, ripening) family of genes are expressed only in plants that interact with ABRE elements as a regulatory mechanism of ABA dependent pathways under stress [18, 19]. The major domain is of Pfam family which is an ABA/WDS domain. This group of genes is a part of regulating complex wherein they play a major role. Particularly in processes involving metabolism of sugars like in fruit ripening, maturation of pollen, senescence and differential responses to various abiotic stresses like drought, salinity, reduced light intensity, cold [20–25]. The protein products of this gene act like chaperons and help in preventing thawing and freezing type denaturation [26]. ASR genes are expressed in different organs *viz*., potato tubers [27], and fruits of tomato, apricot and pomelo [28], pollen of lily [29], leaves and roots of tomato, rice, maize and pine [30,31]. All ASR genes known till date have a DNA binding activity at the N-terminus that is sequence specific and dependent on Zn^2+^ with a nucleus localization signal at the C terminus. Sub-cellular fractionation studies also proved that ASR protein occur in the nucleus and cytoplasm. The potential role of ASR1 gene in drought tolerance in common bean was studied and strong selection pressures, lower gene diversity was found in the accessions [32]. Transgenic *Arabidopsis* with over-expressed ASR gene showed an increase in tolerance to drought and salt and decrease in sensitivity on exposure to exogenous ABA [33]. ASR3 gene was identified as a putative candidate gene for association mapping for tolerance to drought in rice [34]. So far, the characterization studies on ASR genes in chickpea are limited.

Huge genome sequence information available online in public domains (http://www.ncbi.nlm.nih.gov) have served as excellent sources for identification of important genes for insect resistance, quality traits, resistance to different abiotic stresses *viz*., drought, salinity, heat. Such computational studies are valuable in areas of comparative genomics and have enabled us to identify and characterize chickpea ASR genes. In the present study, single ASR homologue was identified in chickpea. Analysis of conserved domains, phylogenetic relationships, three dimensional structure prediction and validation, and functional partners within the query sequence identified a hypothetical protein NP_001351739.1, potentially involved in drought tolerance. The drought responses and relative expression levels of chickpea ASR genes under different treatments were also studied. Our study gives an idea about the role of ASR genes in drought tolerance in chickpea and also indicates similarities with the already characterized proteins which may be possibly used in improvement of chickpea and related pulse crops.

## Results

### Relative water content, chlorophyll, protein content and membrane stability index in control and water stressed plants

**Fig 1:**
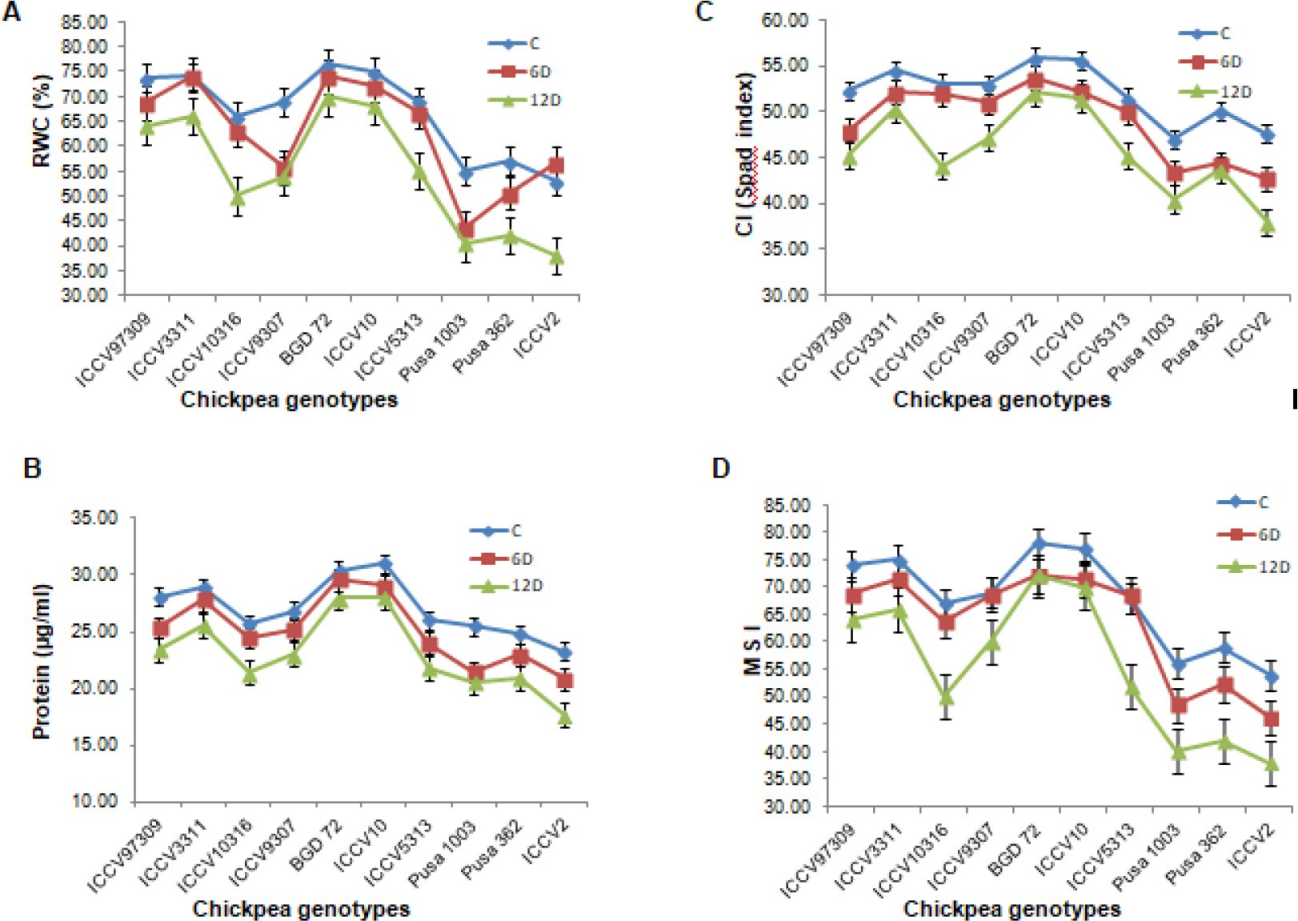
Drought responses of selected chickpea genotypes measured after different periods of stress (0, 6 and 12 days after drought stress treatment). Changes in relative water content (leaf) (A), Protein content (leaf) (B), Chlorophyll index (CI), MSI (D) of selected genotypes of chickpea. Samplings were done at 0day (control), 6^th^ day and 12^th^ day after imposition of drought stress. All the measurements were recorded in three replications and mean values were plotted against the selected chickpea genotypes.

Ten genotypes were used to examine drought responses at different time points *viz*., control (0day), 6day and 12day as defined for chickpea [35]. Susceptible checks (ICCV2, Pusa 1003 and Pusa 362) were also included in analysis for comparative study. Variations in relative water content (RWC,%), chlorophyll index (CI, Spad Units), protein content (μg/ml) and membrane stability index (MSI, mS/cm) were measured to test their responses for improved drought tolerance and better understand their resistance mechanisms, presented in Table 1. There was a significant decrease in all the parameters measured from day 0–12 of drought stress in all the genotypes (Table 2). Control plants maintained a higher RWC (%), CI, protein content and MSI in comparison to drought stressed plants (Fig 1A–1D). Reduction in RWC (%) ranged from 8% to 28.3% in all the genotypes at 12^th^ day after imposing drought stress (Fig 1A). Maximum decrease in RWC (%) was 28.3% in ICCV2; however, BGD72 (8.73%), ICCV10 (9.29%) and ICCV3311 (10.81%) maintained the RWC (%) under drought stress (Table 2 and Fig 1A). Drought stress affected the CI significantly, and the decrease was noteworthy in ICCV2 (20.31%) (Fig 1B). On the contrary, resistant genotypes BGD72 (6.77%), ICCV10 (7.5%) and ICCV3311 (7.57%), retained higher CI at 12^th^ day of drought stress treatment (Table 2 and Fig 1C). Our results also signified a substantial decrease in soluble protein content (leaf) in all the chickpea genotypes under drought stress (Fig 1B). Though, BGD72 showed minimal decrease in protein content (7.89%) over the control, this decrease was prominent in ICCV2 (24.3%) (Table 2 and Fig 1B). Decline in Membrane stability index (MSI) was prominent in all the genotypes on imposition of drought stress (Fig 1D). BGD72 (7.69%), ICCV10 (9.09%) and ICCV3311 (12%) showed minor decrease in MSI under stressed conditions in comparison to control conditions. Conversely, the susceptible genotypes *viz*., ICCV2 (29.62%), Pusa362 (29.15%) and Pusa1003 (28.57%) displayed a much higher reduction in MSI at 12^th^ day of drought stress treatment (Table 2 and Fig 1D). Significant changes observed from control to stressed samples demonstrate stress at morpho-physiological and biochemical levels in all the genotypes.

**Table 1:** ANOVA for all the four physiological traits under study viz., relative water content (RWC, %), chlorophyll index (CI, Spad units), leaf protein content (μg/ ml), membrane stability index (MSI, mS/cm) in the ten chickpea genotypes under drought stress conditions. Measurements were taken at 0 day (control conditions), 6^th^ day after imposition of drought stress, 12^th^ day after imposition of drought stress.

*Source of Variation*	df	RWC	CI	Protein	MSI	*F crit*
**Treatment**	2	376.428**	99.351**	40.72**	386.881**	3.354
**Error**	27	111.155	15.891	8.771	114.133	
**F value**		3.386	6.251	4.642	3.389	

** Significance at p≤0.05

**Table 2:** Mean values of relative water content (RWC, %), leaf protein content, chlorophyll index (CI, Spad units) and membrane stability index (MSI, mS/cm) of the ten chickpea genotypes under drought stress conditions.

	Physiological parameters							
Genotype	RWC (%)		Protein (leaf) (μg/ml)		CI (Spad units)		MSI (mS/cm)	
	C	12D	C	12D	C	12D	C	12D
ICCV97309	73.80^a^	64.00^a^	28.10^a^	23.40^a^	52.30^a^	45.30^a^	74.00^a^	64.20^a^
ICCV3311	74.00^a^	66.00^b^	28.93^a^	25.53^b^	54.50^b^	50.36^b^	75.00^b^	66.00^b^
ICCV10316	66.00^b^	50.00^c^	25.69^b^	21.36^c^	53.10^c^	44.02^c^	67.00^c^	50.00^c^
ICCV9307	69.00^c^	54.00^d^	26.85^c^	22.96^d^	52.95^a^	47.11^d^	69.00^d^	60.00^d^
BGD 72	76.70^d^	70.00^e^	30.40^d^	28.00^e^	55.96^d^	52.16^e^	78.00^e^	72.00^e^
ICCV10	74.97^e^	68.00^f^	30.99^e^	27.00^e^	55.65^d^	51.46^f^	77.00^f^	70.00^f^
ICCV5313	69.00^c^	51.00^g^	26.05^f^	21.83^c^	51.53^e^	45.13^a^	68.00^g^	52.00^g^
Pusa 1003	55.00^f^	40.50^h^	25.48^g^	20.53^f^	47.00^f^	40.46^g^	56.00^h^	40.00^h^
Pusa 362	57.00^g^	42.00^i^	24.78^h^	20.95^f^	50.06^g^	43.66^h^	59.00^i^	41.80^i^
ICCV2	51.00^h^	38.00^j^	23.29^i^	17.62^g^	47.60^f^	37.93^i^	54.00^j^	39.00^j^
**Mean**	66.64	54.35	27.06	23.03	52.07	45.76	67.70	55.50
**CV (%)**	84.51	144.67	6.22	11.11	9.53	21.55	76.46	159.04

Means followed by different letters within a column are significantly different from each other according to Tukey’s Studentized Range (HSD) test at p≤0.05; C: control conditions; 12D: 12^th^ day after imposition of drought stress; CV: coefficient of variation.

### Quantitative real-time PCR (qRT-PCR) analysis

**Fig 2:**
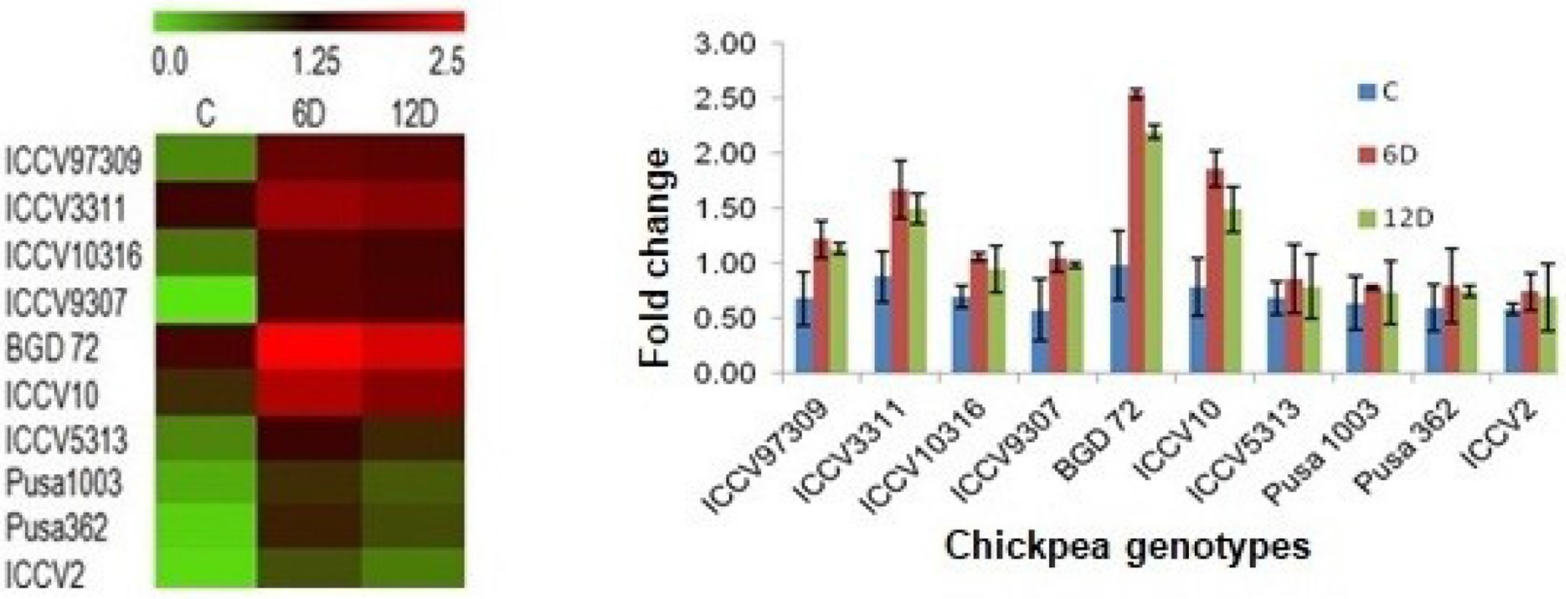
Differential expression of chickpea ASR genes under drought stress conditions. (A) Heat-map showing differential expression of chickpea ASR genes during drought stressed periods (0day, 6^th^ day and 12^th^ day) in selected genotypes of chickpea. The scale at the top represents log_2_ fold change, maximum value is displayed as dark red and minimum value is displayed as light green. (B) Real-time PCR analysis to validate the differential expression of chickpea ASR genes during drought conditions. The Beta Actin gene was used as a reference. Expression was measured after 0, 6 and 12 days after imposition of stress. In control, expression was recorded on day 0 of stress. The mean fold change in ASR gene expression at each time point was calculated using the 2^−ΔΔCT^ method where ΔΔCT = (C_T_,_Target_-C,_Actin_)_Time x_—(C_T_,_Target_-C,_Actin_)_Time 0_. Data are means ± SD of triplicate samples.

To analyze the expression pattern of ASR gene under drought stress in selected genotypes of chickpea, real time quantitative PCR was performed. The Beta Actin gene was used as the reference gene. Samplings were done in triplicates at each time point (0day, 6^th^ day and 12^th^ day after drought stress treatment). The mean fold change in the ASR gene, normalized to Beta Actin gene at different time points was calculated by Ct (cycle threshold) values (S1 Table of S1 File). Normalization with Actin gene produced more consistent and similar results in drought susceptible (ICCV2, Pusa1003 and Pusa362) and tolerant (BGD72, ICCV10 and ICCV3311) genotypes that clearly illustrated differential expression of transcripts of *ASR* gene (Fig 2A). In general, with the induction of drought stress, the expression of ASR gene was up-regulated in all the chickpea genotypes over controls and reached maximum at 6^th^ day, followed by a slight decrease at 12^th^ day. Expression patterns revealed high and significant expression of Abscissic acid and stress ripening gene (ASR) in the drought tolerant genotypes (BGD72, ICCV10 and ICCV3311) in comparison to the susceptible genotypes (ICCV2, Pusa1003 and Pusa362) indicating its sensitivity to drought stress treatment (Fig 2B). Highest relative expression value was observed for BGD72 (2.54 fold higher than the control) at 6^th^ day after imposition of stress. The increase in ASR gene expression was pronounced in ICCV10 (up to 1.86-fold change, 6^th^ day) and ICCV3311 (1.67-fold change, 6^th^ day) followed by a small decrease in fold change to 1.49 by 12^th^ day of drought stress. Decreased relative expression level was evident in ICCV2 (0.69-fold change) at 12^th^ day after stress imposition. No significant differences in the relative expression level of ASR were observed from control and treated samples in Pusa1003 and Pusa362, though the induction was higher in ICCV97309, ICCV9307 and ICCV10316.

### Amplification of ASR genes from chickpea

**Fig 3:**
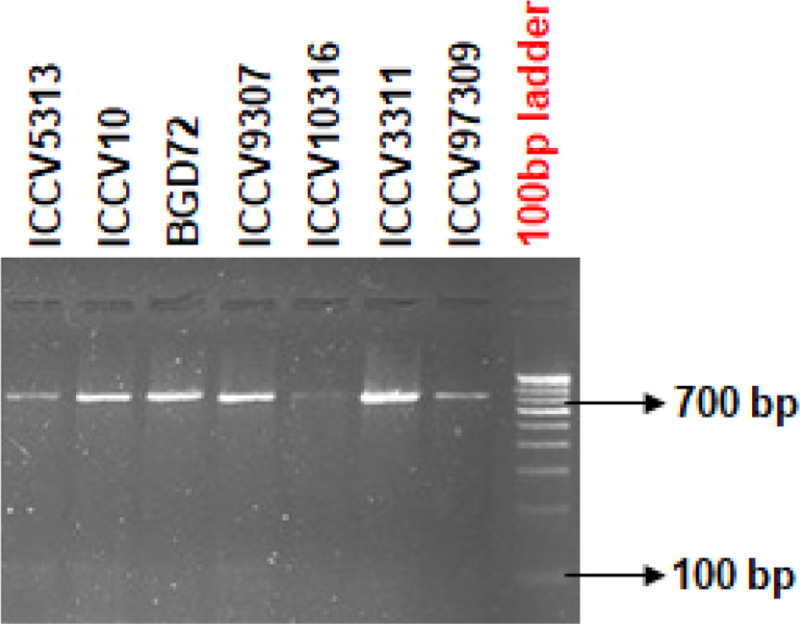
PCR Amplification of seven chickpea genotypes using ASR gene specific marker. PCR amplification of seven chickpea genotypes *viz*., ICCV97309, ICCV3311, ICCV10316, ICCV9307, BGD72, ICCV10, and ICCV5313 was done using ASR gene specific primer and revealed a single amplicon ranging from 680-700bp; Marker-100 bp Banglore Genei DNA ladder.

**Fig 4:**
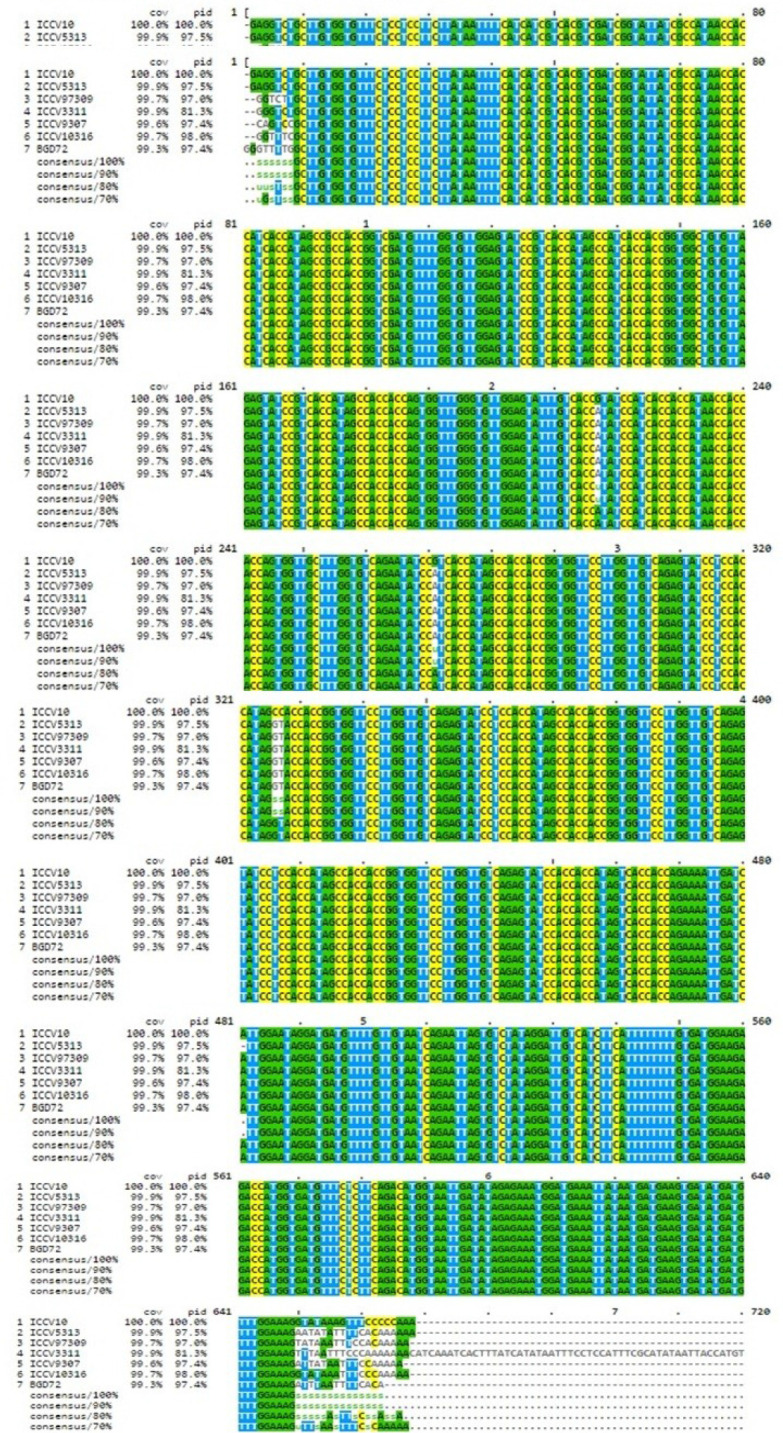
Jalview of multiple sequence alignment of ASR gene homologues of seven chickpea genotypes. Conserved region was observed in all the chickpea genotypes with very minor variations (www.ebi.ac.uk).

The ASR gene homologues (Fig 3) were isolated from *Medicago* gene sequence available at NCBI EST database (DbEST-http://www.Ncbi.nm.nih.gov/dbEST/) [36]. Sequences of the ASR genes ranged from 680bp to 700bp nucleotides. Conserved region was observed in all the chickpea genotypes with very minor variations (Fig 4). BLASTn results confirmed 99.32% similarity with chickpea putative ABA/WDS induced protein (LOC101493413), mRNA with E value of 0.00.

### Molecular analysis of chickpea ASR homologue with other legume plants

**Fig 5:**
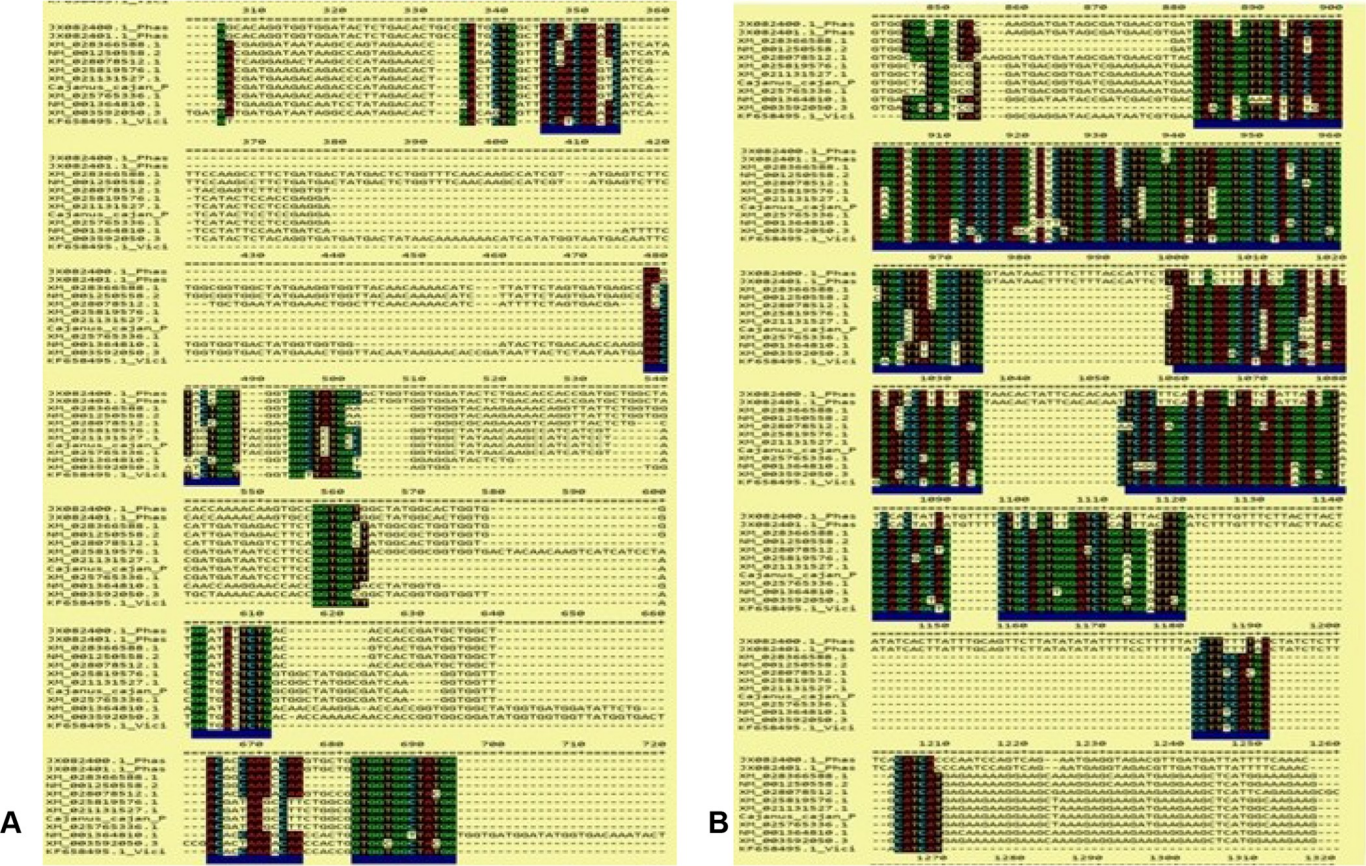
a. Jalview of chickpea ASR gene with different legumes ASR genes. Multiple sequence alignment of ASR gene was done using MUSCLE software and revealed conserved nucleotides at various positions. b. Jalview of chickpea ASR gene with different legumes ASR genes Contd. Multiple sequence alignment of ASR gene was done using MUSCLE software revealing conserved nucleotides at various positions.

Comparison of the chickpea ASR homologue with other legume plants available at NCBI database revealed conserved nucleotides at various positions (Fig 5A–5B). ‘A’ at position number 336, 345, 350, 351, 478, 606, 664, 669, 670, 691, 883, 886, 892, 895, 898, 900, 901, 904, 907, 909, 910, 916, 918, 928, 931, 940, 953, 999, 1008, 1017, 1019, 1023, 1029, 1032, 1061, 1063, 1064, 1068, 1071, 1076, 1107, 1191, 1205, 1208. ‘T’ at position number 340, 497, 499, 608, 610, 683, 690, 894, 926, 934, 942, 943, 947, 950, 959, 966, 972, 973, 1000, 1067, 1079, 1098, 1103, 1108, 1110, 1118, 1184, 1185, 1206. ‘G’ at position number 341, 484, 485, 494, 495, 500, 602, 603, 611, 682, 684, 685, 687, 688, 693, 694, 850, 882, 885, 888, 903, 906, 908, 927, 929, 936, 937, 939, 945, 946, 948, 951, 957, 963, 969, 974, 1003, 1034, 1065, 1078, 1102, 1106, 1120. ‘C’ at position number 346, 349, 354, 609, 665, 673, 912, 915, 917, 930, 949, 955, 958, 960, 970, 1014, 1082, 1085, 1097, 1100, 1109, 1183, 1204, 1207 (S2 Table of S1 File).

### Neighbour joining analysis

**Fig 6:**
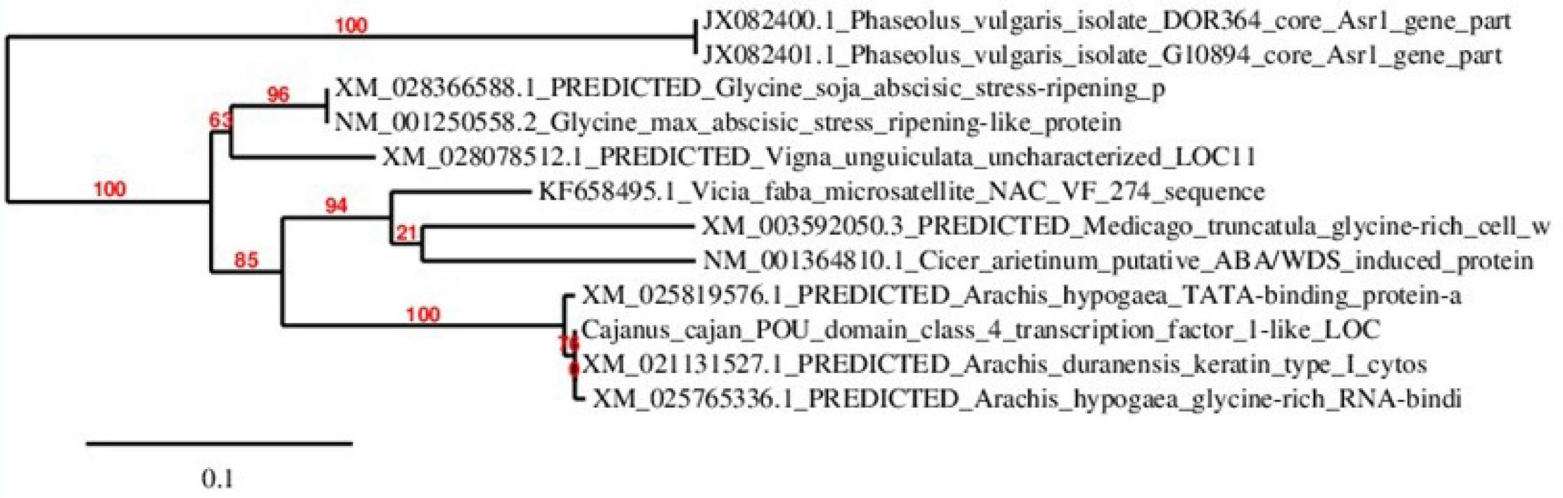
Phylogenetic tree of chickpea ASR gene with ASR genes in different legumes. Phylogenetic tree was constructed by Neighbour joining method with 1000 replications in bootstrap test using Treedyne software (https://www.phylogeny.fr/).

Multiple sequence alignment of ASR gene was done using MUSCLE software and a phylogenetic tree was constructed by Neighbour joining method with 1000 replications in bootstrap test using Treedyne software. The *Cicer arietinum* gene encoding putative ABA/WDS domain containing protein was grouped with *Medicago trancatula* glycine-rich cell protein encoding gene with bootstrap value 21 and *Phaseolus vulgaris* Asr genes, *Arachis hypogea* glycine rich TATA-binding protein encoding genes and *Cajanus cajan* POU domain class 4 transcription factor-1 gene with 100 bootstrap value. The *Glycine soja*, *Glycine max* Asr genes were grouped closer to *Vigna ungiculata* and *Vicia faba* sequences (Fig 6)

### Target protein sequence analysis

**Fig 7:**
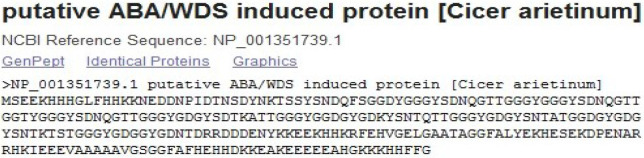
Target protein sequence downloaded from NCBI (http://www.ncbi.nlm.nih.gov). Blastx revealed 99.44% similarity with putative ABA/WDS induced protein (*Cicer arietinum*) with accession ID NP_001351739.1.

Blastx results showed 99.44% similarity with putative ABA/WDS induced protein (*Cicer arietinum*) with accession ID NP_001351739.1 and E value 7e-22, the sequence of which was downloaded from NCBI database (Fig 7). The protein was reported from *Cicer arietinum* with 257 amino acids and has been found to encode a family of plant proteins induced by water deficit stress or abscisic acid (ABA) stress and ripening.

### Structural analysis of the protein NP_001351739.1

**Fig 8:**
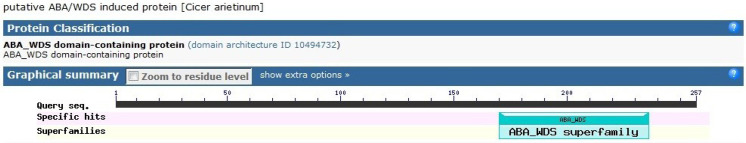
Conserved domains in chickpea ASR gene homologue. NCBI tool for conserved domain search (CDD) identified ABA-WDS domain-containing protein and ABA_WDS superfamily induced by water deficit stress.

Conserved domains NCBI CDD tool identified pfam02496 ABA/WDS induced protein and one superfamily ABA_WDS with an E-value of 9.03e-22 (Fig 8). Expasy Protparam tool assessed different characteristics of the predicted protein. The molecular weight of the predicted protein was found to be 27.1 KDa and isolelectric point 5.05. This domain comprised mainly of glycine (G) and asparagine (D) with 22.6% and 9.3% respectively. The atomic composition of ABA-WDS domain with formula C_1151_H_1661_N_343_O_428_S_1_ and 3584 atoms showed presence of 25 positively charged residues (Arg + Lys) and 46 negatively charged residues (Asp + Glu). The instability index of 27.62 indicated stability of the ABA-WDS domain induced protein.

### Alignment and neighbour joining analysis

**Fig 9:**
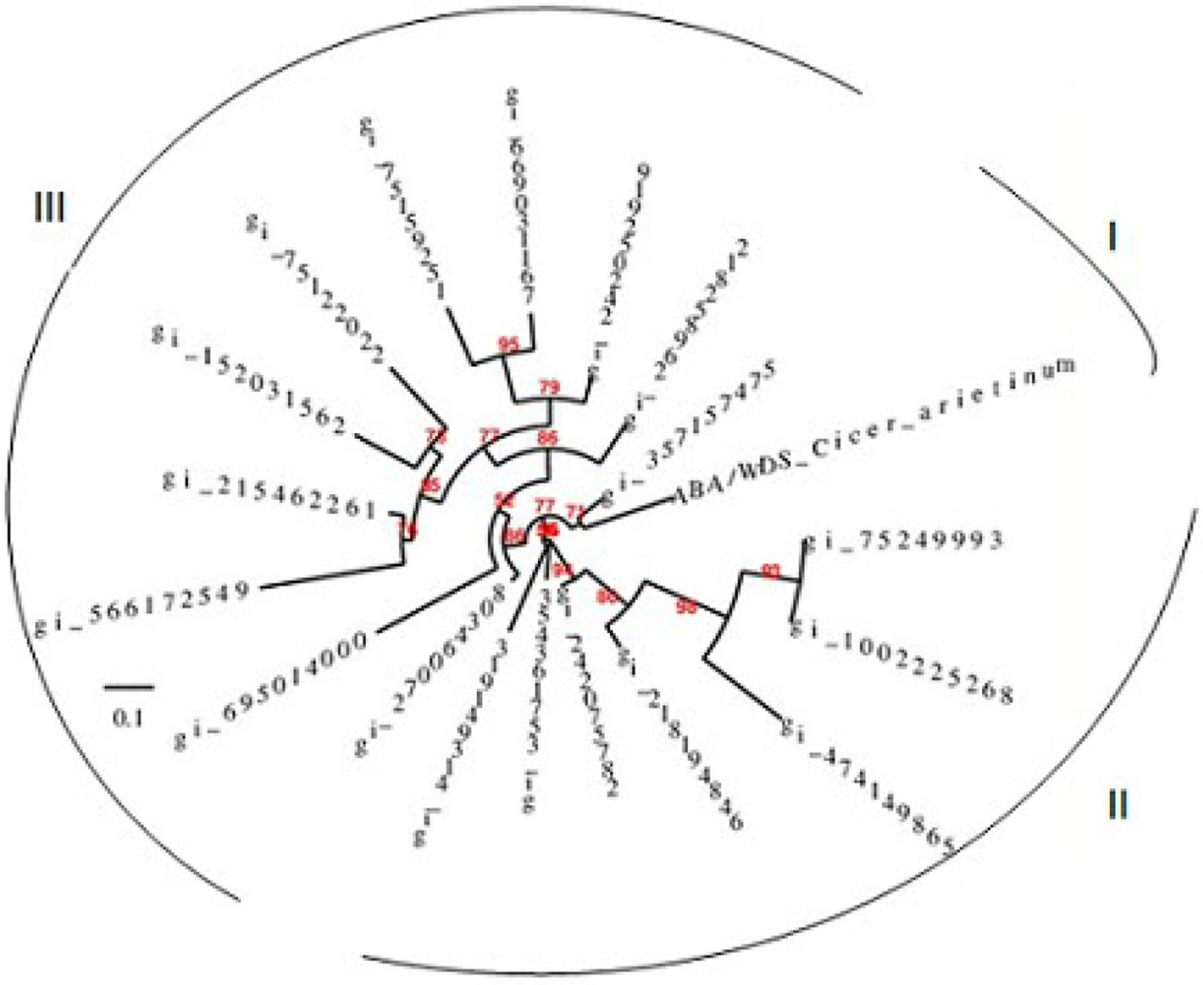
Phylogenetic analysis of the conserved ABA/WDS domain induced protein with those of model plants available in NCBI database. Sequence alignments were performed using phylogeny.fr web service and the circular phylogenetic tree was constructed using the Tree Dyne software (https://www.phylogeny.fr/).

Alignment of conserved ABA/WDS domain induced protein and phylogenetic tree was constructed using Neighbour joining analysis (Fig 9). The phylogram divided the ASR proteins into three major clusters. Multiple sequence alignment showed *Cicer arietinum* ABA/WDS induced protein belonging to cluster I grouped with *Brachypodium distachyon* abscissic stress ripening protein 3 with boostrap value 71. Closely related ABA/WDS induced proteins from other crop plants in the cluster II included *Triticum uratu* abscissic stress ripening protein with bootstrap value 98, putative bundle sheath specific protein_1_Os01g0963600 and homeotic protein female sterile protein *Oryza sativa Japonica* group with boostrap value 93, hypothetical protein_Os1_15903_ *Oryza sativa indica* group with boostrap value 80, putative fruit ripening protein_Os01g0959100_protein and unnamed protein_*Triticum aestivum* with bootstrap value of 95.

### Structural prediction and validation

**Fig 10:**
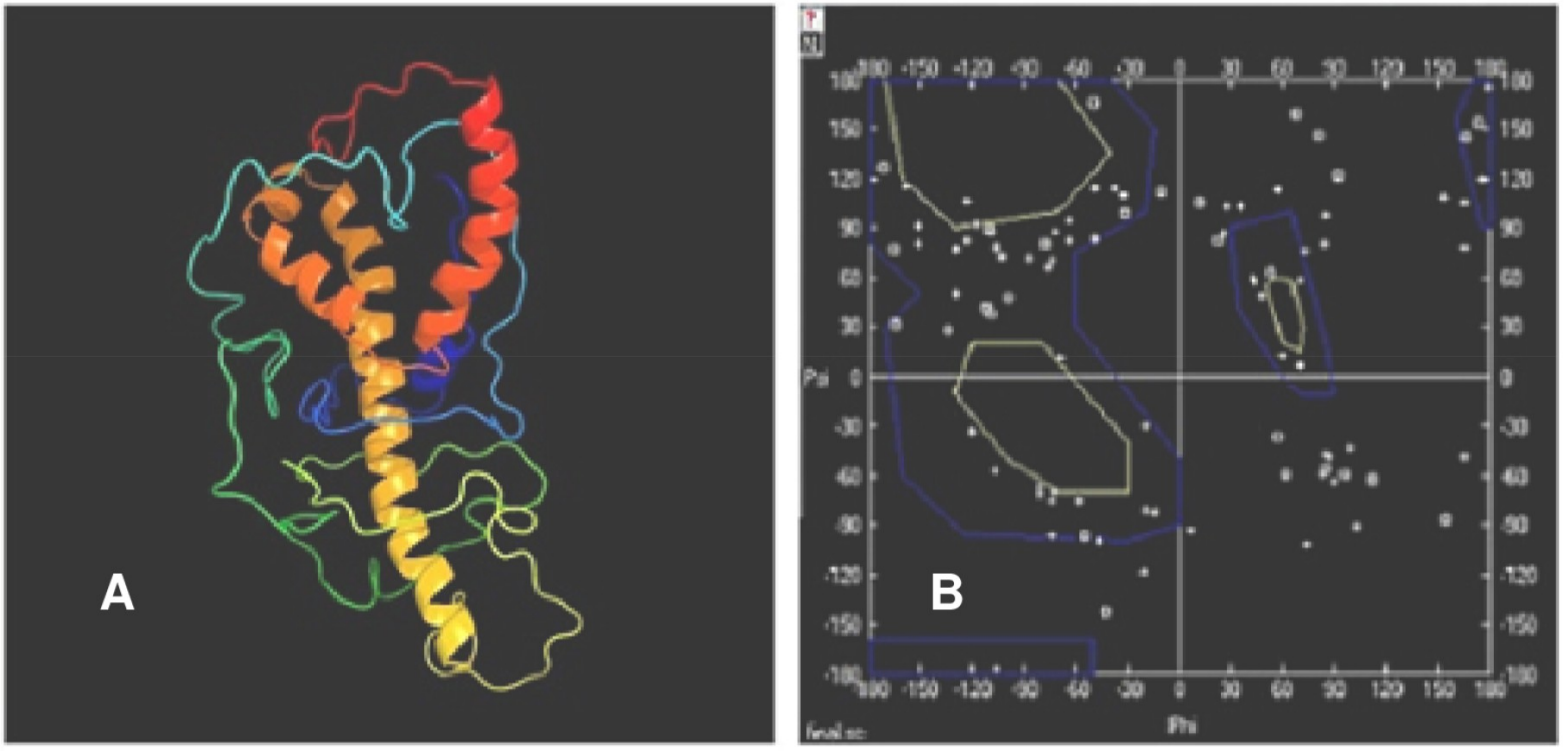
Structure modelling of the hypothetical protein NP_001351739.1. **(a)** Structure prediction of ABA/WDS domain containing protein constructed by using Phyre2 (http://www.sbg.bio.ic.ac.uk/~phyre2/html/page.cgi?id=index); **(b)** Structure validation (Ramachandran Plot) of ABA/WDS domain containing protein through Swiss-Pdb Viewer v4.1.0.

**Fig 11:**
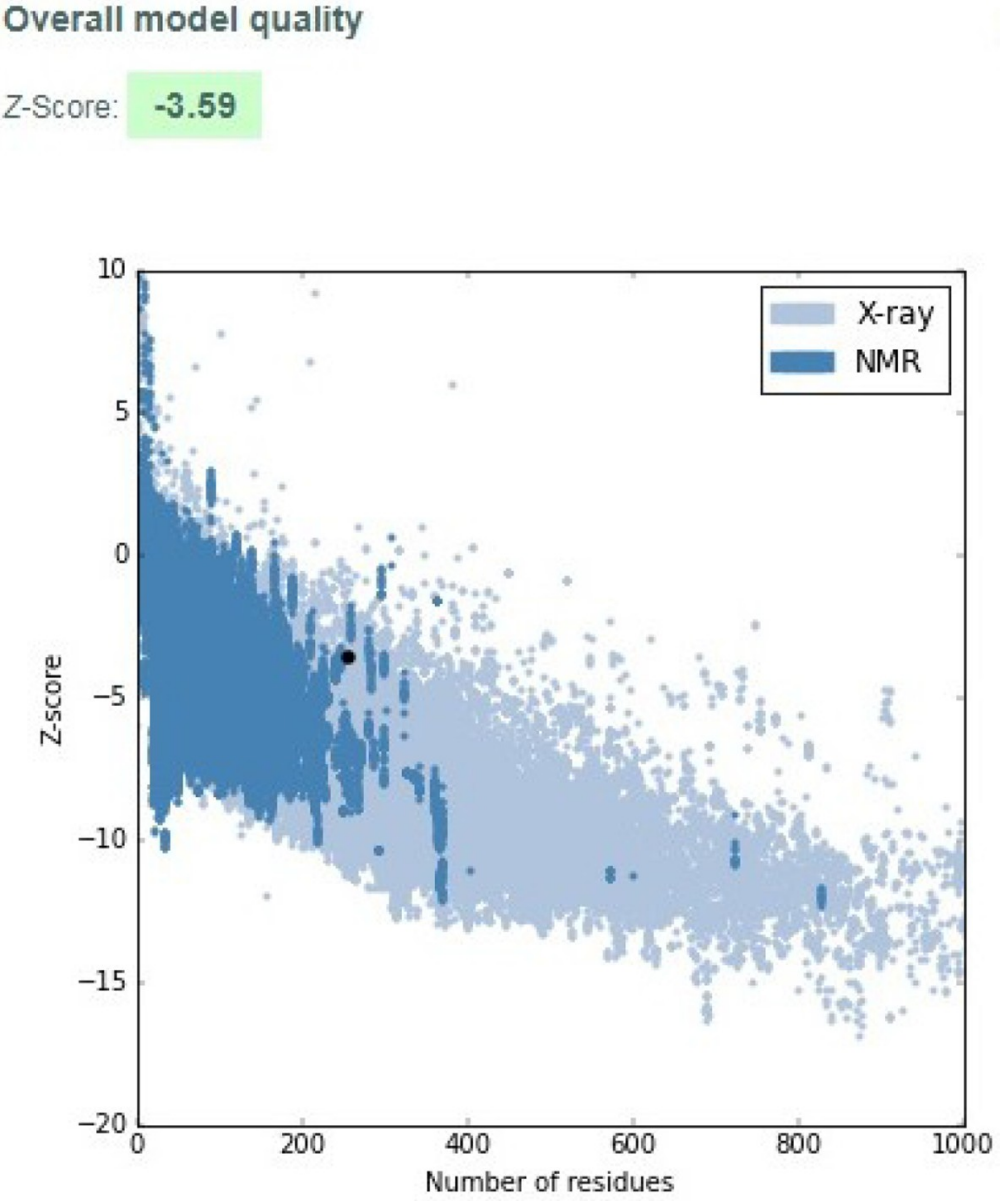
Protein structure analysis (ProSA) of the target protein NP_001351739.1. ProSA tool z-scores computed by NMR spectroscopy (indicated in dark blue) or X-ray crystallography (indicated in light blue) with regard to length. The z-score is indicated by with a black dot.

**Fig 12:**
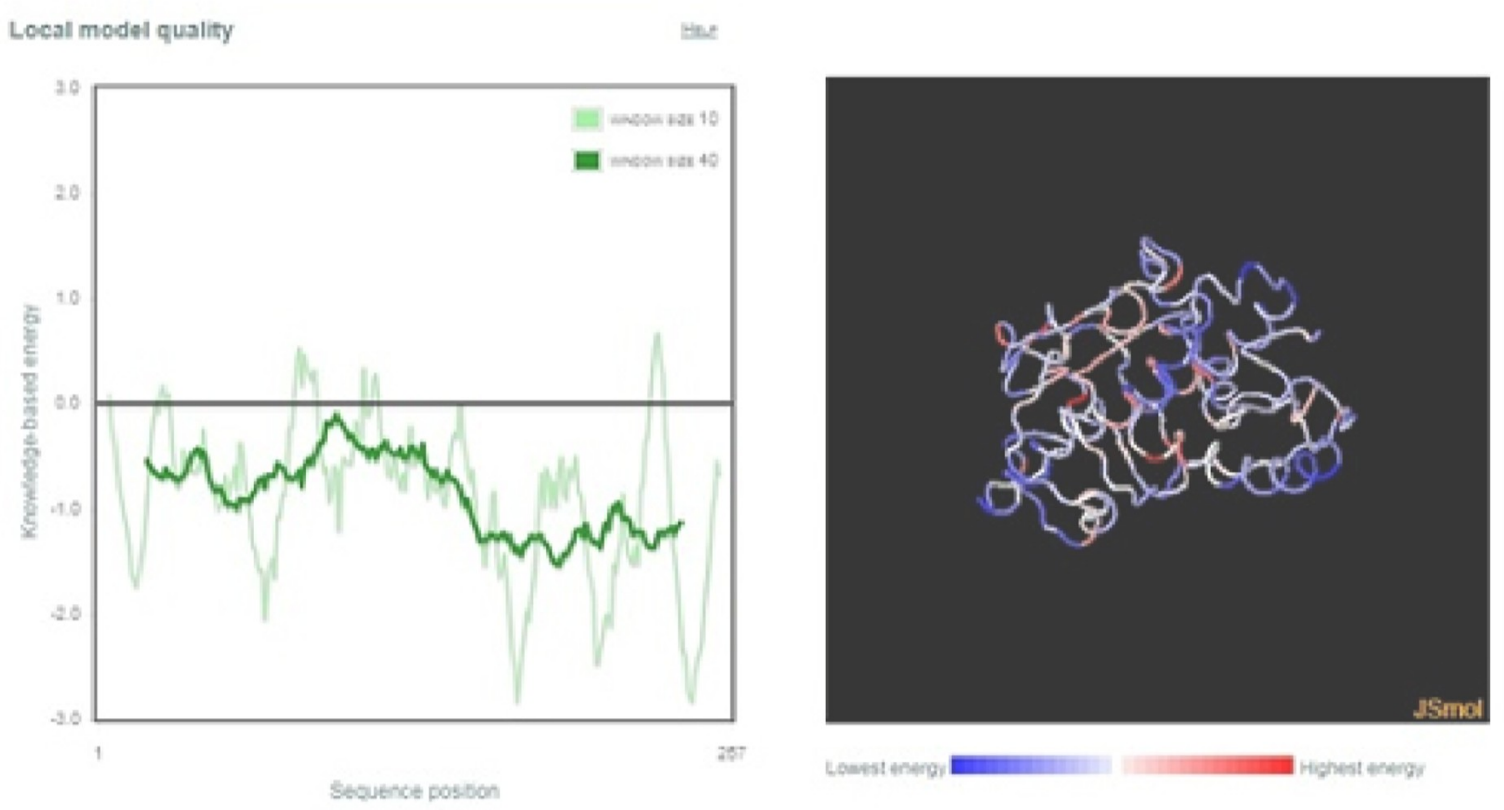
Energy plot of the target protein NP_001351739.1. **(a)** Erroneous parts of the model; **(b)** Regions that contribute to the overall bad score of the predicted model indicating the local model quality.

The three-dimensional model of the hypothetical protein was constructed using Phyre2 database (Fig 10A). The results revealed that the protein comprised of only alpha helixes and had no β-sheets. Alpha helixes accounted for 35% of the total protein. The Psi-Phi plot showed that 72.2% amino acid with 184 residues were present in the most favored regions, 14.5% amino acids with 37 residues in the allowed regions and 13.3% amino acids with 34 residues in the disallowed regions (Fig 10B). ProSA-web revealed that the z-score of the protein was -3.59 and within the range of scores found for proteins of similar size (Fig 11). The energy plot indicating the energies as a function of amino acid position was also plotted (Fig 12A). The parts of the predicted model that contribute to the overall bad score were also indicated (Fig 12B).

### STRING analysis

**Fig 13:**
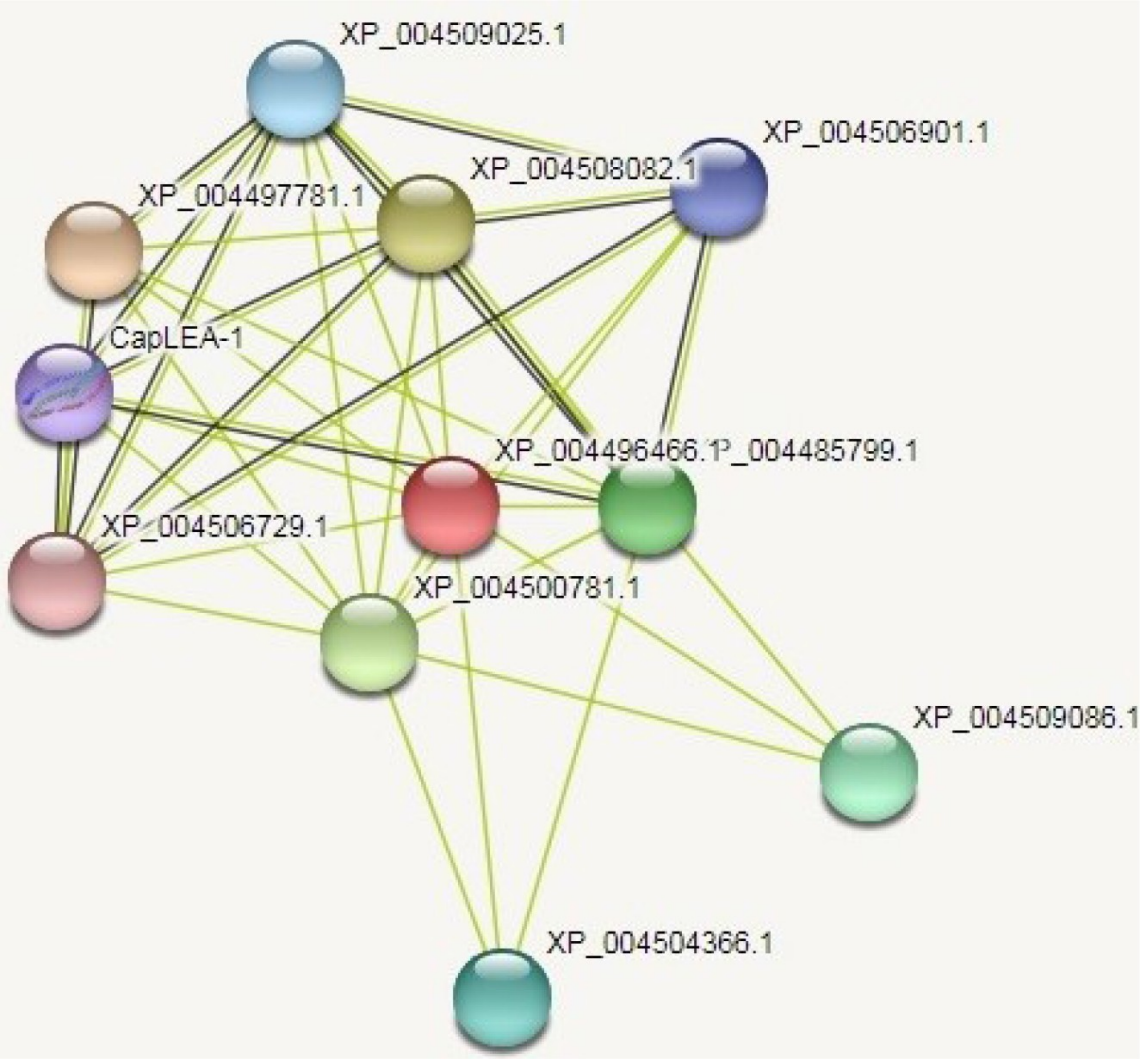
Protein-protein interactions of the target protein NP_001351739.1. STRING network analysis was done to study the interactions of our target protein (indicated in red) with other proteins (http://string-db.org/) for its functioning.

**Fig 14:**
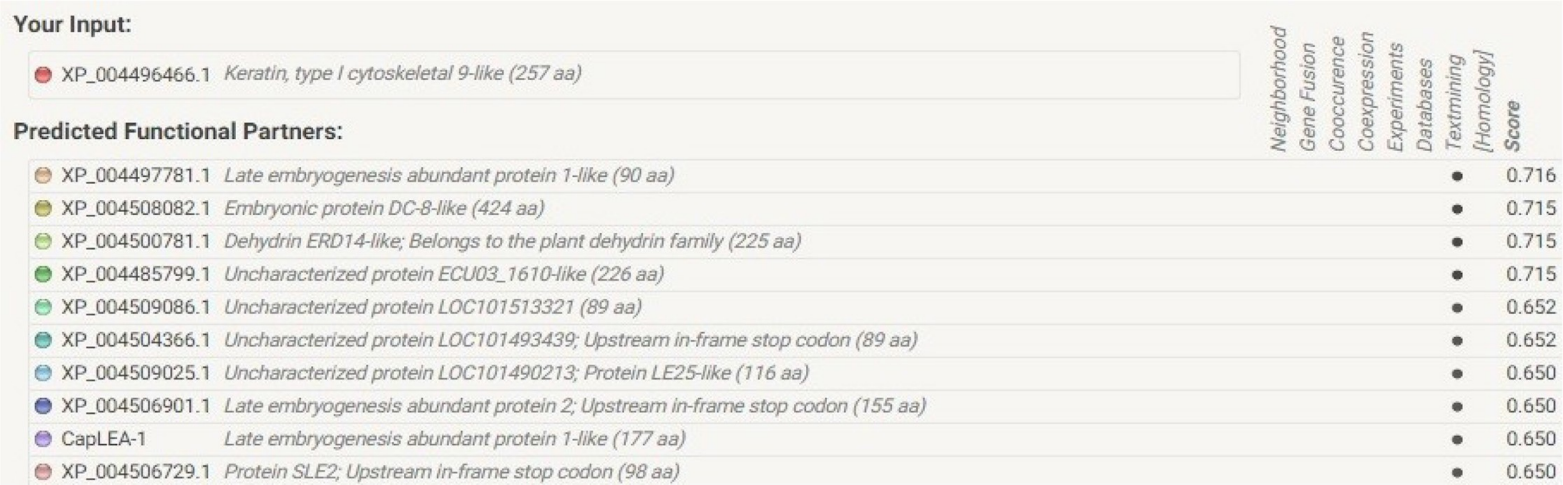
Predicted functional partners of the target protein NP_001351739.1. Functional partners required for its functioning of the target protein were predicted using STRING database signifying its role in drought tolerance.

Protein-protein interactions of the hypothetical protein NP_001351739.1 were analyzed using STRING database and the network obtained is shown in Fig 13. The network analysis revealed our target protein interacts with ten different proteins for carrying out its functions (Fig 14).

## Discussion

Pulses have a great potential to improve human health as a rich source of protein, soil health through nitrogen fixation and helps in attaining food and nutritional security. In recent years pulses are consistently coming under the centre stage of research focus because of its importance. The up swinging prices of pulses and the nutritional importance have forced the policy makers to pay attention towards pulses particularly in the vegetarian population and increasing vegetarian community worldwide. Year 2016 has been declared by the UNO as the “International year of Pulses”. There has been a reduction in the pulse availability per person per day in the last 50 years from 70 grams/capita/day to 34 grams/capita/day (http://www.faostat3.fao.org). The major challenges impeding the pulse production and productivity are narrow base in the cultivated varieties, genotype and environment interaction, multiple biotic and abiotic stresses, difficulty in screening and precisely identifying the target traits [37].

Chickpea with nutritionally essential components and lesser anti-nutritional factors has the best composition among legumes [38]. Chickpeas are the major source of protein in vegetarian society [4]. Chickpea is also called as gram or Bengal gram in common language. It is self-pollinated crop with out-crossing rate less than 1%. Two chickpeas desi (dark coloured seed coat) and kabuli (white coloured seed coat) are known having varied gene pool [39]. Chickpea productivity remained stagnated and low since many years partly because of numerous environmental stresses and insufficient genetic variability in various traits due to the domestication process [40]. Drought is one of the major abiotic stresses affecting the crop productivity all over the world and chickpea like several other legumes is highly susceptible to terminal drought stress. Reduction in yield parameters has been linked to the adverse effects of drought stress on growth processes of plants *viz*., cell growth, biomass, leaf area index and plant yield. Plants survive the environmental stresses and overcome the harmful effects of drought stress with the help of numerous mechanisms. Phenomics assisted with genomic approaches appear to be a dependable solution to decipher these mechanisms and identify solutions for combating drought at a fast pace and improving yield [41]. The present study describes comprehensively the isolation and characterization of ASR gene. The studies on this gene and its molecular function in drought tolerance are very limited in chickpea.

In the present study, seven chickpea genotypes were selected to examine drought responses at 0day, 6^th^ day and 12^th^ day after stress treatment and variations in their physiological parameters were assessed for speedy characterization of their drought tolerance (Table 1). All the chickpea genotypes showed a significant decline in RWC(%), CI, protein content and MSI under stressed conditions in comparison to control conditions (Table 2 and Fig 1A–1D). RWC was considered as the best measure for water status of a plant in mid 80s as it indicates the balance between water absorbed and consumed through transpiration. Under stressed conditions, reduction in RWC has been established at various stages of growth in chickpea *viz*., seedling stage, early flowering and podding. Higher retention of water in tolerant genotypes under stress has been confirmed by many workers [42, 43]. Under drought stress, significant reduction in RWC (%) was observed in all the genotypes at different time points in comparison to controls (Table 2 and Fig 1A). Per cent decrease in RWC was found to be highest in ICCV2 (28.3%) followed by Pusa1003 (26.36%). BGD72 (8.73%), ICCV10 (9.29%) and ICCV3311 (10.81%) maintained a considerably higher RWC (%) in both control and stressed conditions enabling them to perform better in terms of physiological processes under stress. In contrast, ICCV2 (28.3%), Pusa1003 (26.36%) and Pusa362 (26.31%) showed maximum decrease in RWC signifying their vulnerability to drought stress. MSI indicates the cell membrane damage by measuring electrical conductivity of cell leachates under drought. Membrane injuries and leakage of electrolytes from the membrane triggers programmed cell death in plants and also assist in remobilization for seed development [44]. Reduction in membrane stability index (MSI) at different stages under moisture stress has also been confirmed in earlier studies [42]. The per cent decrease in MSI in the chickpea genotypes under stressed conditions ranged from 7–30% (Table 2 and Fig 1D). Many workers have also reported severe influence of drought stress on membrane thermo-stability, canopy temperature depression and yield traits mainly filled pods and seeds per plant [45]. Increased temperature under drought stress damages the cell walls and increases leakage of electrolytes [44, 46]. Relatively lesser decrease in MSI was observed in BGD72 (7.69%), ICCV10 (9.09%) and ICCV3311 (12%) with higher RWC (%) signifying their tolerance to drought. RWC and MSI have been suggested for screening the germplasm for drought tolerance [2]. Drought stress also hinders photosynthetic machinery of plants by bringing about changes in the chlorophyll content [47]. Modification in total chlorophyll, phenolics and proteins of the plants may govern their drought tolerance [48, 49]. Significant reduction was observed in chlorophyll content of all the selected chickpea genotypes under stressed conditions. The percent decrease in chlorophyll index under stress ranged from 6–21%. The tolerant genotypes BGD72 (52.16), ICCV10 (51.46) and ICCV3311 (50.36) maintained high SPAD values in contrast to Pusa362 (43.66), Pusa1003 (40.46) and ICCV2 (37.93) that showed significant decrease under stressed conditions (Table 2 and Fig 1C). Diversity studies among 43 lentil genotypes confirmed significant variations for twelve different phenotypic traits and positive correlation between stable lines and SPAD index establishing use of chlorophyll index as a standard measure for tolerance to drought [50]. Considerable reduction of soluble proteins has been seen in chickpea varieties *viz*., Bivaniej and ILC482 and Pirouz under stressed conditions [51]. Remarkable changes in quantity and quality of soluble proteins under stress have been detected in chickpea [52]. The percentage reduction in soluble protein content (leaf) ranged from 7–25% in the selected genotypes of chickpea. Maximum soluble protein was found in BGD 72 (28 mg/ml) followed by ICCV10 (27mg/ml) and ICCV3311 (25.53mg/ml) having higher SPAD values whereas minimum protein was found in ICCV2 (17.63 mg/ml) followed by Pusa 362 and Pusa 1003 (20.96 mg/ml and 20.53 mg/ml, respectively) with lower SPAD values (Table 2 and Fig 1B). Progressive increase in water stress significantly decreased net photosynthesis rate and protein content in moong bean genotypes [53].

PCR amplicons were then isolated from seven chickpea cultivars and sequenced by gene specific markers [36] using an ABI3500xL genetic analyser (Applied Biosystems, USA). These gene sequences were subjected to sequence alignment using MUSCLE software. The chickpea ASR homologue was compared with other legumes and the conserved nucleotides were identified. Jalview results proved that all the ASR nucleotide sequences in comparison with other legumes show evidence of variations at various positions in the sequence region and the conserved sequence region comprising possibly the ABA/WDS domain (**Fig 5A and 5b**). The phylogenetic tree showing relatedness with other legumes was generated using Treedyne software by neighbor joining method. *Cicer arietinum* gene encoding putative ABA/WDS domain containing protein mRNA showed high similarity with *Phaseolus vulgaris* Asr genes, *Arachis hypogea* glycine rich TATA-binding protein encoding genes and *Cajanus cajan* POU domain class 4 transcription factor-1 gene with maximum bootstrap value 100. *Glycine soja*, *Glycine max* Asr genes were grouped in a different cluster closer to *Vigna ungiculata* and *Vicia faba* sequences (**Fig 6**) with high bootstrap values. Similar findings confirmed that *Cicer arietinum* gene family was closely related to *Medicago tranculata* while soybean, pigeonpea and common bean sharing a common ancestor grouped in a separate cluster [54].

The changes in the expression of chickpea ASR gene relative to the β-actin gene at different points (control, 6^th^ day and 12^th^ day after drought stress treatment) were studied using qRT-PCR. Results confirmed that drought stress significantly increased the ASR gene expression that possibly elicited increased responsiveness towards drought tolerance in chickpea. Expression patterns revealed high and significant expression of ASR gene in all the genotypes under drought stress. Expression of ASR gene was prominent in BGD72, ICCV10 and ICCV3311 at 6^th^ day of stress compared with controls with a slight decrease at 12^th^ day of stress, however, there was no major change in its expression in ICCV2, Pusa1003 and Pusa362 under stressed conditions. It has been proposed ASR1 gene expression increases under stress in a variety of species [55].The results of qRT-PCR analysis of OsASR1 and OsASR3 expression revealed that drought stress mainly regulates the expression of all ASR genes in rice and their over-expression in transgenic plants improved their drought and cold stress tolerance. Transgenic *Brachypodium distachyon* L. plants over-expressing BdASR4 gene retained more water and displayed higher tolerance to drought and antioxidant activities in comparison to the wild plants [56]. Physiological studies also proved the involvement of ASR1 gene in stress tolerance in transgenic tobacco, tomato, maize and rice [57].

The ASR gene sequences were confirmed at protein level using BlastX and Expasy server (http://expasy.org/cgi-bin/protparam). BlastX results showed 100 per cent similarity with chickpea hypothetical ABA/WDS induced protein (NP_001351739.1) with highest score of 486 and lowest Evalue of 0.00. The conserved domain search tool in NCBI identified a pfam02496 ABA/WDS induced protein and an ABA/WDS superfamily with a low E-value of 9.03e-22. Expasy tool revealed that the ABA/WDS domain of the 27.1KDa predictive protein (pI 5.05) was primarily composed of 25 positively charged residues and 46 negatively charged residues. The instability index value of 27.62 also confirmed that the predicted protein is stable. All ASR proteins largely have Glu, Ala, His, Lys and Gly residues and a continuous ABA/WDS domain [58].

Tomato ASR1 protein with DNA binding activity and pI 7.3 is a chromatin bound protein that interacts directly with DNA or indirectly by interacting with other proteins. ASR1 proteins may be nuclear or may be dispersed in the cytoplasm. In general, 40-60KDa molecules diffuse passively through nuclear pores depending on their concentration gradient, whereas other molecules are transported actively [59] through the nuclear localization signals. These NLS (rich in basic amino acids) are recognized by certain docking molecules *viz*., receptor molecules at the nuclear pore [60]. Sub-cellular localization studies proved ASR1 being smaller in size can pass through the nuclear pore with ease and contains a signal sequence KK**DA**KK**EE**KKK**L**R near the C-terminus [26]. Maize ASRs also have a Zn-binding domain at the N-terminal and a nuclear targeting signal with two abscissic acid/water deficit stress domains (ABA/WDS) at the C-terminal. Hybrid assays and sub-cellular fractionation studies confirmed the role of ZmASR proteins as transcription factors or molecular chaperons in different plant species [61].

Multiple sequence alignment of the chickpea conserved ABA/WDS domain containing protein (NP_001351739.1) with those of model plants available in NCBI was also done using Treedyne software and a phlogenetic tree was generated using neighbour joining method. The *Cicer arietinum* ABA/WDS induced protein grouped with *Brachipodium distachyon* ASR protein3 with bootstrap value 71. The *Cicer arietinum* ASR protein was found to be closely related to monocotyledonous Gramineae species, *Zea mays* and *Oryza sativa*, *Sorghum bicolor* in comparison to other plant species including cottonwood, banana species. Similar phylogenetic relationships were found while comparing the apple ASR genes with those of other plant species [58].

Three-dimensional structure of the hypothetical protein NP_001351739.1 was predicted using homology modeling in Phyre2 database (http://www.sbg.bio.ic.ac.uk/~phyre2/html/page.cgi?id=index). The protein comprised of alpha helixes accounting for 35% of the total protein (Fig 10A). ASR proteins adapt two different conformations *viz*., alpha helical or a polyproline type II under different environmental stresses. Polyethylene glycol (PEG) and glycerol stabilize the α-helical conformation in general while lower temperature, lower pH and increased NaCl stabilize the PII conformation. This structural plasticity is critical for plant stress resistance, facilitating their response to drought and interaction with target proteins [62]. The predicted model was then evaluated through Ramachandran plot using Swiss-Pdb Viewer v4.1.0 program by plotting the Psi-Phi angles of the amino acid residues against each other. The Psi-Phi pairs had 72.2% residues in the most preferred regions, 14.5% residues in allowed regions and 13.3% residues in outlier regions as shown in **Fig 10B**.

Protein Structure Analysis (ProSA) tool identifies the regions that contribute to an overall bad score in the predicted model and has long been used for their refinement and validation. The energy plots and the Z-scores indicate the problems in the predicted structure of the target protein. The z-score of NP_001351739.1 was found to be -3.59 indicating the high quality of the predicted protein model as shown in **Fig 11**. Energy plot of NP_001351739.1 is shown in **Fig 12A**. The positive values indicate the erroneous parts of the model in general. Residual error plots indicating reliability of the local model were also plotted and visualized by color gradients. Blue colored regions indicate more reliable regions and red regions indicate the probable unreliable regions. Models that slide towards blue regions from light red color are considered to be of high quality (**Fig 12B**).

Software packages available online offer great opportunities for analyzing biological systems. AraNet, GeneMania, and STRING being user-friendly [63–65] have been used for studying the protein interactions, regulatory networks, gene associations and their biological pathways [66–68]. STRING database was used to predict the direct and indirect interactions of NP_001351739.1 (http://string-db.org/). The hypothetical protein was found to interact with ten different proteins (Fig 13) for its functioning *viz*., XP_004497781.1 late embryogenesis abundant protein 1-like (90 amino acid), XP_004508082.1 embryonic protein DC 8-like (424 amino acid), XP_004500781.1 Dehydrin ERD 14 like (225 amino acid), XP_004485799.1 uncharacterized protein ECU09_1610-like (225 amino acid), XP_004509086.1 uncharacterized protein LOC101513321 (89 amino acid), XP_004504366.1 uncharacterized protein LOC101493439; upstream in-frame stop codon (89 amino acid), XP_004509025.1 uncharacterized protein LOC101490213, protein LE25-like (116 amino acid), XP_004506901.1 late embryogenesis abundant protein 2; upstream in-frame stop codon (155 amino acid), CapLEA-1 late embryogenesis abundant protein 1-like (177 amino acid) and XP_004506729.1 protein SLE2; upstream in-frame stop codon (98 amino acid). The predicted functional partners of the hypothetical protein NP_001351739 confirm its role in drought tolerance in chickpea (**Fig 14**). Integrated high-throughput approaches employing molecular networks with phenomics together may provide assumptions and address precise biological queries [69].

Drought QTLs have been identified using different approaches *viz*., QTL mapping [70], sequence similarity based candidate gene allele diversity analysis [36] and genome wide association study (GWAS) [71] by some workers. Ten genes were found to express under abiotic stresses [36]. Functional validation was also done for these genes using the already reported genes from model plants. Linkage analysis and association mapping approach was also used to identify and validate the chickpea genes and QTLs for moisture stress tolerance based on sequence similarity approach [72]. Candidate genes identified may be used to develop cultivars with desired tolerance to drought and ensure greater genetic gains and also enhance the probability of breeding widely adapted high yielding hybrids in chickpea. ASR is the most widely reported drought stress responsive gene [73]. The ASR gene family has evolved from Spermatophyta. ASR gene is regulated by water deficit, salinity stress and hormone Abscisic acid (ABA), low temperature and intensity of light [74]. ASR genes were first recognized in tomato [75] and consequently, in different plant species *Zea mays* (nine), *Sorghum bicolor* (seven), *Oryza sativa* (six), *Brachypodium distachyon* (six), *Pinus taeda* (four), *Phaseolus vulgaris* (two) and *Vitis vitifera* (one) [76,77,18]. Transgenic studies confirmed ASR genes could be involved in ABA signalling pathways enabling the plants to respond to external stresses [78,79] and transgenic plants with over-expressed ASR gene were found to be more tolerance to water and salt stress [26]. During late embryogenesis accumulation of tomato ASR1 was observed. Electrophoretic assays and direct visualization also confirmed formation of homodimers in DNA by ASR1 in response to water stress [23, 80]. In contrast, activity of tomato ASR2 promoter was enhanced in response to ABA in papaya and tobacco, while reduction was observed in tomato and potato [81]. Transgenic *Arabidopsis* lines over-expressing maize ASR genes exhibited better growth performance and higher survival rates as compared to wild type under drought conditions. These lines had lower malondialdehyde content and higher ABA and proline content improving their drought tolerance. The results thus, proved Zm ASR3 enhance drought tolerance via an ABA dependent pathway [82]. Their precise role in conferring improved tolerance to drought and salt has also been established in tomato, rice and lily [33, 34, 26]. Consequently, reports on involvement of ASR gene in legumes in drought responses are insufficient.

## Material and methods

### Experimental material, soil selection, drought stress treatment

Seven promising genotypes of chickpea (*Cicer arietinum*) *viz*., ICCV97309, ICCV3311, ICCV10316, ICCV9307, BGD72, ICCV10, and ICCV5313 were selected from Pulse Research Laboratory, Division of Genetics, Indian Agricultural Research Institute (IARI), Pusa, New Delhi. The amalgamated soil (peat to vermiculite, 1:1) with pH 7.6 and conductivity 0.4 ds/m was taken from the IARI field and each genotype was sown in 13cm diameter plastic pots in three replications under glasshouse conditions at the National Phytotron Facility, Indian Agricultural Research Institute, New Delhi (28°08’N 77°12’) with a photoperiod of 12h in a completely randomized design (CRD) design in the year 2019–20. The temperature was maintained at 24°C in the day and 18°C in the night. These pots were irrigated with 200 ml water on daily basis. Drought stress was imposed on 12 day old plants by withholding water for 6 days and 12days respectively [35]. Control plants were watered regularly for the same duration. Leaf tissues of the control and drought stressed plants were collected at different time points *viz*., 0d (control), 6d and 12d and fixed in liquid nitrogen and stored at -80°C for RNA isolation. These genotypes were identified on their relative basis of tolerance to drought [83].

### Determination of relative water content

Relative water content (RWC) of selected chickpea genotypes was measured at different time points (0 day, 6^th^ day and 12^th^ day) as per the standard method [84]. Young leaf tissues of chickpea were collected and their fresh weights were recorded. The leaf tissues were then incubated in petriplates containing distilled water for 4 hours for calculating their turgid weights. Oven drying of the leaves was done for 72 hours at 60°C and then the plant dry weights were recorded. RWC was calculated using the following formula:

RWC(%)=(FW-DW/TW-DW)*100

Where, FW- Fresh weight; DW—Dry weight; TW—Turgid weight

### Estimation of chlorophyll index and protein content

Konica Minolta SPAD 502 Plus chlorophyll meter was used for measuring the chlorophyll index of the selected chickpea genotypes at three different time points (0 day, 6^th^ day and 12^th^ day). For estimating protein content (leaf) at different time points, crushing of leaves was done in 50mM phosphate buffer with pH 7.8. The protein content was estimated by colorimetric method [85]. Absorbance of the samples was recorded on Beckman DU^®^ 640 spectrophotometer at 595 nm [86]. The Bovine Serum Albumin (Sigma, USA) was used as a standard. The protein content was expressed in μg ml^-1^.

### Determination of membrane stability index

400 mg fresh leaf sample was taken and added to test tubes containing 10ml of distilled water. The test tubes were kept in a water bath maintained at 45°C for 30 minutes and conductivity (C1) was noted using a portable conductivity meter. These test tubes were again placed in water bath maintained at 100°C for 10 minutes and then conductivity was noted again (C2) [87]. The MSI was calculated using formula:

MSI=1-(C1/C2)*100

### Statistical analysis

The data for all physiological parameters were subjected to standard method of statistical analysis such as analysis of variance (ANOVA) using XLSTAT software. The mean values and coefficient of variation (CV) were calculated for each parameter. The standard errors of the mean were presented in the figures as error bars. The mean comparisons were performed using Tukey’s Studentized Range (HSD) test. The Tukey’s Studentized Range (HSD) test at p = 0.05 was employed to test the differences among the treatment means for the measured parameters at 0 (control), 6^th^ day and 12^th^ day after imposing drought stress.

### RNA extraction and quantitative real-time PCR (qRT-PCR) analysis

RNA isolation was done by using NucleoZOL (Takara Bio). Genomic DNA and other contaminants were removed by precipitation. One phase RNA extraction was followed by conversion to first strand cDNA using the Accuscript high fidelity cDNA synthesis kit (Agilent). Brilliant III ultra fast SYBR Green was used to measure the relative changes in the expression of chickpea ASR genes under water stressed conditions. The cDNA from leaf tissues were used as template. The Beta Actin gene was used as the reference gene. The qRT-PCR was conducted on a CFX 96 Real Time PCR (Biorad) in a reaction volume of 25μL, that comprised of 2μL chickpea samples cDNA, 0.5 μL each ASR specific forward and reverse primer, 12.5 μL Brilliant III ultra fast SYBR Green QPCR master mix (Agilent), and 9.5 μL nuclease-free molecular biology grade water. The qRT-PCR reaction cycle included 95°C for 3 min followed by 40 cycles at 95°C for 5 s, 60°C for 12s. The relative expression levels of chickpea ASR genes under different treatments were then calculated using the 2^−ΔΔCT^ method [88].

### DNA extraction and PCR amplification

The genomic DNA of seven chickpea genotypes was extracted from young leaves using the CTAB method [39]. Purified DNA was used for PCR amplification using ASR gene specific forward 5’-GGGAACTAATCCTTTCCAAACA-3’ and reverse 5’-CTGCAGCACCTAACTCACCA-3’ primer custom synthesized by G-Biosciences, USA [83]. PCR was carried out in the Chickpea Molecular Breeding Laboratory, Division of Genetics, ICAR-IARI using a G-STORM thermal cycler (Labtech, France). The PCR master mix comprised of 20ng of the template DNA, 1.6μl of 10X Tris borate-ethylenediamine-tetra acetic acid (TBE), 1μl of 10mM dNTP mix (Genei, Banglore), 1μl each of 5μM forward and reverse primer and 0.3μl of 3U Taq Polymerase (Genei, Banglore). The PCR cycling reaction consisted of three steps, starting with initial denaturation (90°C for 3min) followed by 38 cycles of denaturation (94°C for 20sec), annealing (55°C for 50sec) and elongation (72°C for 50sec) followed by final elongation (72°C for 7mins) [89]. The PCR amplicons were visualized on a 3% 1X TBE buffer using 100bp DNA ladder (Thermo Scientific, USA). The gel image was documented using UV light gel documentation system (UVITECH Imaging System, UK).

### Nucleotide sequence analysis

PCR amplicons of the seven chickpea genotypes were purified using BigDye terminator v3.1 kit (Applied Biosystems, USA) and were sequenced using an ABI3500xL genetic analyser (Applied Biosystems, USA). Sequencing data was analyzed in Sequencing Analysis v 5.4. Raw sequences were assembled using the forward and reverse sequences of each genotype in KB v 1.4.1.8(KB base caller) tool. The assembled chickpea ASR gene sequence was submitted to NCBI GenBank (MK937569) using the web-based submission tool Sequin from the NCBI home page (https://www.ncbi.nlm.nih.gov/Sequin/). A comparison BLAST tools (blastn) were used determine the sequence identities of the assembled sequence (http:// www.ncbi.nlm.nih.gov). Sequence alignments were performed using MUSCLE software and phylogram of *Cicer* ASR gene with ASR gene sequences of different legumes available at NCBI database was constructed by neighbor joining method.

### Computational protein analysis

The ASR sequences were also verified at the protein level using blastx and Expasy server (http:// expasy.org/cgi-bin/protparam) and their conserved domains were identified using the NCBI tool for conserved domain search (CDD). Common characteristics of the predicted protein including molecular weight, isoelectric point (pI), amino acid composition, aliphatic and instability index were assessed using protparam tool. The amino acid sequences for the ABA/WDS conserved domain from different crop plants were retrieved from NCBI CDD tool. Sequence alignments were performed and a circular phylogenetic was constructed using Treedyne software (https://www.phylogeny.fr/). The three dimensional structure was predicted by homology modeling using the Phyre2 database and validated with Swiss-PDB Viewer (SPDBV). The target protein model was further refined by the Program structure Analysis (ProSA) program that predicts the structure of the target protein using the molecule viewer Jmol in order to find out the regions that contribute to errors in the protein model. Network analysis was done using the STRING database (http://string-db.org/.) to find out functional and physical interactions of the predicted protein.

### Conclusion

Present study reveals that increased expression of ASR gene under drought stress possibly enabled the tolerant chickpea genotypes to perform better under stressed conditions. The results show a close relationship between qRT-PCR data and physiological characterization of the genotypes under drought stress conditions which displayed higher RWC(%), MSI, CI and protein content in BGD72, ICCV10 and ICCV3311 in comparison to the susceptible genotypes ICCV2, Pusa1003 and Pusa362. Modifications have occurred at various nucleotides in gene sequence of ASR genes during evolution. The NCBI CDD tool, Expasy Protparam, Phyre2, Swiss PDB viewer and ProSA tool revealed important features *viz*., primary structure, secondary structure, z-scores of hypothetical protein NP_001351739. NP_001351739 with Arginine, lysine, glutamic acid, Asparagine and ABA/WDS conserved domain that might comprise the nuclear localization signals (NLS) and pass through the nuclear pores inducing different plant stress inducible genes. The predicted functional partners identified by STRING network analysis also proved that NP_001351739 interacts with various LEA proteins, dehydrins for its functioning and is likely to play an important role in drought tolerance in chickpea. This hypothetical ASR protein might have enhanced the ASR gene activity as a transcription factor mediating drought responses in chickpea. This study could be useful in identification of new ASR genes that play a major role in drought tolerance and also develop functional markers for chickpea improvement.

## Supporting information

S1 File (PDF)

S1 Raw image PCR Amplification of seven chickpea genotypes using ASR gene specific marker. PCR amplification of seven chickpea genotypes *viz*., ICCV97309, ICCV3311, ICCV10316, ICCV9307, BGD72, ICCV10, and ICCV5313 was done using ASR gene specific primer and revealed a single amplicon ranging from 680-700bp; Marker-100 bp Banglore Genei DNA ladder.
(PDF)
