# Supplementary material for: Characterization of ASR gene and its role in drought tolerance in chickpea (Cicer arietinum L.)
Source: PLoS One. 2020 Jul 14;15(7):e0234550. doi: 10.1371/journal.pone.0234550 (PMC7360048; doi:10.1371/journal.pone.0234550)
Supplement: S1 File — (PDF) [file pone.0234550.s001.pdf]

S1 Table. Relative expression of Abscissic acid and stress ripening gene in control and drought stressed samples of chickpea. Beta actin gene was used as reference. The fold change in expression of ASR gene was studied after 0, 6th and 12th day of drought stress. Samples collected in triplicates at every time point. In control, expression was recorded on day 0 of drought stress treatment. 2^-ct indicates the mean fold change in ASR gene expression relative to the control samples.

Sample	Time (day)	Ct value	Average Ct	STD DEV	B.ACT Ct value	Average Ct	STD DEV	^ CT (BACT)	2^-ct B.ACT
		31.70	31.88	0.26	31.09	31.33	0.21	0.55	0.68
		32.22			31.60				
1	0	31.73			31.30				
		32.00	31.98	0.23	31.99	31.72	0.30	0.17	0.89
		32.10			31.30				
2	0	31.57			31.87				
		30.95	30.81	0.10	29.90	30.29	0.50	0.52	0.70
		30.77			30.99				
3	0	30.71			29.98				
		32.46	32.12	0.28	30.98	31.33	0.28	0.79	0.58
		31.77			31.67				
4	0	32.12			31.33				
		32.07	31.68	0.31	31.19	31.66	0.38	0.03	0.98
		31.30			32.13				
5	0	31.66			31.65				
		31.78	31.46	0.26	30.98	31.11	0.10	0.35	0.78
		31.14			31.23				
6	0	31.46			31.11				
		31.96	31.78	0.15	31.30	31.23	0.06	0.55	0.68
		31.60			31.16				
7	0	31.77			31.22				
		32.71	32.42	0.24	31.60	31.78	0.14	0.64	0.64
		32.12			31.95				
8	0	32.44			31.80				
		31.31	31.06	0.21	29.70	30.33	0.50	0.73	0.60
		30.79			30.92				
9	0	31.10			30.38				
		31.99	31.95	0.05	30.60	31.20	0.49	0.76	0.59
		31.98			31.79				
10	0	31.89			31.20				

1	6	31.37	31.13	0.17	31.35	31.42	0.05	-0.29	1.22
		31.02			31.45				
		30.99			31.45				
2	6	30.99	30.62	0.26	30.80	31.23	0.31	-0.61	1.52
		30.38			31.38				
		30.49			31.50				
3	6	33.80	33.85	0.04	33.90	33.94	0.03	-0.09	1.06
		33.89			33.98				
		33.86			33.93				
4	6	30.79	30.95	0.13	31.94	31.02	0.76	-0.07	1.05
		31.11			30.09				
		30.93			31.03				
5	6	31.61	31.65	0.04	33.01	32.99	0.02	-1.35	2.54
		31.69			32.97				
		31.66			33.00				
6	6	32.64	32.56	0.16	33.69	33.45	0.33	-0.90	1.86
		32.33			32.99				
		32.70			33.68				
7	6	31.19	30.81	0.31	30.65	30.59	0.05	0.22	0.86
		30.44			30.52				
		30.8			30.60				
8	6	31.38	31.35	0.02	31.12	31.00	0.10	0.36	0.78
		31.32			30.87				
		31.36			31.00				
9	6	31.03	31.44	0.34	30.33	31.11	0.64	0.33	0.80
		31.85			31.89				
		31.44			31.11				
10	6	31.03	30.84	0.17	30.13	30.41	0.23	0.43	0.74
		30.63			30.70				
		30.85			30.40				
1	12	30.40	30.34	0.05	30.57	30.52	0.05	-0.18	1.13
		30.27			30.45				
		30.35			30.53				
2	12	30.87	30.75	0.14	30.94	31.33	0.29	-0.58	1.49
		30.56			31.65				
		30.82			31.40				
3	12	30.86	30.64	0.21	30.48	30.54	0.05	0.08	0.95
		30.36			30.59				
		30.70			30.56				
4	12	30.16	30.13	0.03	30.09	30.11	0.01	0.02	0.99

		30.08			30.12				
		30.14			30.11				
		30.94	31.02	0.06	32.21	32.16	0.05	-1.14	2.21
		31.08			32.09				
5	12	31.03			32.17				
		31.25	31.04	0.21	31.50	31.62	0.08	-0.58	1.49
		30.76			31.67				
6	12	31.11			31.68				
		31.07	31.44	0.29	31.02	31.10	0.06	0.34	0.79
		31.79			31.16				
7	12	31.44			31.11				
		31.91	31.54	0.29	30.19	31.09	0.73	0.45	0.73
		31.48			31.99				
8	12	31.22			31.09				
		30.51	30.55	0.05	30.09	30.14	0.05	0.41	0.75
		30.62			30.20				
9	12	30.53			30.13				
		30.80	31.17	0.31	30.53	30.64	0.57	0.53	0.69
		31.15			31.39				
10	12	31.55			30.00				

S2 Table. Conserved nucleotide positions in ASR gene family. Comparison of the chickpea ASR homologue with other legume plants available at NCBI database revealed conserved nucleotides at various positions

	**Conserved consensus nucleotide positions in ASR gene**																
**Accession number**	336	340	341	345	346	349	350	351	354	478	484	485	494	495	497	499	500
NM_001364810.1 l *Cicer arietinum*	A	T	G	A	C	C	A	A	C	A	G	G	G	G	T	T	G
XM_003592050.3 l *Medicago truncatula*	A	T	G	A	C	C	A	A	C	A	G	G	G	G	T	T	G
KF658495.1 l *Vicia faba*	A	T	G	A	C	C	A	A	C	A	G	G	G	G	T	T	G
XM_025819576.1 l *Arachis hypogaea*	A	T	G	A	C	C	A	A	C	A	G	G	G	G	T	T	G
XM_021131527.1 l *Arachis duranensis*	A	T	G	A	C	C	A	A	C	A	G	G	G	G	T	T	G
Cajanus cajan POU domain	A	T	G	A	C	C	A	A	C	A	G	G	G	G	T	T	G
XM_025765336.1 l *Arachis hypogaea*	A	T	G	A	C	C	A	A	C	A	G	G	G	G	T	T	G
JX082400.1 l *Phaseolus vulgaris*	A	T	G	A	C	C	A	A	C	A	G	G	G	G	T	T	G
JX082401.1 l *Phaseolus vulgaris*	A	T	G	A	C	C	A	A	C	A	G	G	G	G	T	T	G
XM_028366588.1 l *Glycine soja*	A	T	G	A	C	C	A	A	C	A	G	G	G	G	T	T	G
NM_001250558.2 l *Glycine max*	A	T	G	A	C	C	A	A	C	A	G	G	G	G	T	T	G
XM_028078512.1 l *Vigna unguiculata*	A	T	G	A	C	C	A	A	C	A	G	G	G	G	T	T	G

**Accession number**	602	603	606	608	609	610	611	664	665	669	670	673	682	683	684	685	687
NM_001364810.1 l *Cicer arietinum*	G	G	A	T	C	T	G	A	C	A	A	C	G	T	G	G	G
XM_003592050.3 l *Medicago truncatula*	G	G	A	T	C	T	G	A	C	A	A	C	G	T	G	G	G
KF658495.1 l *Vicia faba*	G	G	A	T	C	T	G	A	C	A	A	C	G	T	G	G	G
XM_025819576.1 l *Arachis hypogaea*	G	G	A	T	C	T	G	A	C	A	A	C	G	T	G	G	G
XM_021131527.1 l *Arachis duranensis*	G	G	A	T	C	T	G	A	C	A	A	C	G	T	G	G	G
Cajanus cajan POU domain	G	G	A	T	C	T	G	A	C	A	A	C	G	T	G	G	G
XM_025765336.1 l *Arachis hypogaea*	G	G	A	T	C	T	G	A	C	A	A	C	G	T	G	G	G
JX082400.1 l *Phaseolus vulgaris*	G	G	A	T	C	T	G	A	C	A	A	C	G	T	G	G	G
JX082401.1 l *Phaseolus vulgaris*	G	G	A	T	C	T	G	A	C	A	A	C	G	T	G	G	G
XM_028366588.1 l *Glycine soja*	G	G	A	T	C	T	G	A	C	A	A	C	G	T	G	G	G
NM_001250558.2 l *Glycine max*	G	G	A	T	C	T	G	A	C	A	A	C	G	T	G	G	G
XM_028078512.1 l *Vigna unguiculata*	G	G	A	T	C	T	G	A	C	A	A	C	G	T	G	G	G

**Accession number**	688	690	691	693	694	850	882	883	885	886	888	892	894	895	898	899	900
NM_001364810.1 l Cicer arietinum	G	T	A	G	G	G	G	A	G	A	G	A	T	A	A	G	A
XM_003592050.3 l Medicago truncatula	G	T	A	G	G	G	G	A	G	A	G	A	T	A	A	G	A
KF658495.1 l Vicia faba	G	T	A	G	G	G	G	A	G	A	G	A	T	A	A	G	A
XM_025819576.1 l Arachis hypogaea	G	T	A	G	G	G	G	A	G	A	G	A	T	A	A	G	A
XM_021131527.1 l Arachis duranensis	G	T	A	G	G	G	G	A	G	A	G	A	T	A	A	G	A
Cajanus cajan POU domain	G	T	A	G	G	G	G	A	G	A	G	A	T	A	A	G	A
XM_025765336.1 l Arachis hypogaea	G	T	A	G	G	G	G	A	G	A	G	A	T	A	A	G	A
JX082400.1 l Phaseolus vulgaris	G	T	A	G	G	G	G	A	G	A	G	A	T	A	A	G	A
JX082401.1 l Phaseolus vulgaris	G	T	A	G	G	G	G	A	G	A	G	A	T	A	A	G	A
XM_028366588.1 l Glycine soja	G	T	A	G	G	G	G	A	G	A	G	A	T	A	A	G	A
NM_001250558.2 l Glycine max	G	T	A	G	G	G	G	A	G	A	G	A	T	A	A	G	A
XM_028078512.1 l Vigna unguiculata	G	T	A	G	G	G	G	A	G	A	G	A	T	A	A	G	A

**Accession number**	901	903	904	906	907	908	909	910	912	915	916	917	918	926	927	928	929
NM_001364810.1 l *Cicer arietinum*	A	G	A	G	A	G	A	A	C	C	A	C	A	T	G	A	G
XM_003592050.3 l *Medicago truncatula*	A	G	A	G	A	G	A	A	C	C	A	C	A	T	G	A	G
KF658495.1 l *Vicia faba*	A	G	A	G	A	G	A	A	C	C	A	C	A	T	G	A	G
XM_025819576.1 l *Arachis hypogaea*	A	G	A	G	A	G	A	A	C	C	A	C	A	T	G	A	G
XM_021131527.1 l *Arachis duranensis*	A	G	A	G	A	G	A	A	C	C	A	C	A	T	G	A	G
Cajanus cajan POU domain	A	G	A	G	A	G	A	A	C	C	A	C	A	T	G	A	G
XM_025765336.1 l *Arachis hypogaea*	A	G	A	G	A	G	A	A	C	C	A	C	A	T	G	A	G
JX082400.1 l *Phaseolus vulgaris*	A	G	A	G	A	G	A	A	C	C	A	C	A	T	G	A	G
JX082401.1 l *Phaseolus vulgaris*	A	G	A	G	A	G	A	A	C	C	A	C	A	T	G	A	G
XM_028366588.1 l *Glycine soja*	A	G	A	G	A	G	A	A	C	C	A	C	A	T	G	A	G
NM_001250558.2 l *Glycine max*	A	G	A	G	A	G	A	A	C	C	A	C	A	T	G	A	G
XM_028078512.1 l *Vigna unguiculata*	A	G	A	G	A	G	A	A	C	C	A	C	A	T	G	A	G

Accession number	930	931	934	936	937	939	940	942	943	945	946	947	948	949	950	951	953
NM_001364810.1 l *Cicer arietinum*	C	A	T	G	G	G	A	T	T	G	G	T	G	T	G	C	A
XM_003592050.3 l *Medicago truncatula*	C	A	T	G	G	G	A	T	T	G	G	T	G	T	G	C	A
KF658495.1 l *Vicia faba*	C	A	T	G	G	G	A	T	T	G	G	T	G	T	G	C	A
XM_025819576.1 l *Arachis hypogaea*	C	A	T	G	G	G	A	T	T	G	G	T	G	T	G	C	A
XM_021131527.1 l *Arachis duranensis*	C	A	T	G	G	G	A	T	T	G	G	T	G	T	G	C	A
Cajanus cajan POU domain	C	A	T	G	G	G	A	T	T	G	G	T	G	T	G	C	A
XM_025765336.1 l *Arachis hypogaea*	C	A	T	G	G	G	A	T	T	G	G	T	G	T	G	C	A
JX082400.1 l *Phaseolus vulgaris*	C	A	T	G	G	G	A	T	T	G	G	T	G	T	G	C	A
JX082401.1 l *Phaseolus vulgaris*	C	A	T	G	G	G	A	T	T	G	G	T	G	T	G	C	A
XM_028366588.1 l *Glycine soja*	C	A	T	G	G	G	A	T	T	G	G	T	G	T	G	C	A
NM_001250558.2 l *Glycine max*	C	A	T	G	G	G	A	T	T	G	G	T	G	T	G	C	A
XM_028078512.1 l *Vigna unguiculata*	C	A	T	G	G	G	A	T	T	G	G	T	G	T	G	C	A

Accession number	955	957	958	959	960	963	966	969	970	972	973	974	999	1000	1003	1008	1014
NM_001364810.1 l *Cicer arietinum*	C	G	C	T	C	G	T	G	C	T	T	G	A	T	G	A	C
XM_003592050.3 l *Medicago truncatula*	C	G	C	T	C	G	T	G	C	T	T	G	A	T	G	A	C
KF658495.1 l *Vicia faba*	C	G	C	T	C	G	T	G	C	T	T	G	A	T	G	A	C
XM_025819576.1 l *Arachis hypogaea*	C	G	C	T	C	G	T	G	C	T	T	G	A	T	G	A	C
XM_021131527.1 l *Arachis duranensis*	C	G	C	T	C	G	T	G	C	T	T	G	A	T	G	A	C
Cajanus cajan POU domain	C	G	C	T	C	G	T	G	C	T	T	G	A	T	G	A	C
XM_025765336.1 l *Arachis hypogaea*	C	G	C	T	C	G	T	G	C	T	T	G	A	T	G	A	C
JX082400.1 l *Phaseolus vulgaris*	C	G	C	T	C	G	T	G	C	T	T	G	A	T	G	A	C
JX082401.1 l *Phaseolus vulgaris*	C	G	C	T	C	G	T	G	C	T	T	G	A	T	G	A	C
XM_028366588.1 l *Glycine soja*	C	G	C	T	C	G	T	G	C	T	T	G	A	T	G	A	C
NM_001250558.2 l *Glycine max*	C	G	C	T	C	G	T	G	C	T	T	G	A	T	G	A	C
XM_028078512.1 l *Vigna unguiculata*	C	G	C	T	C	G	T	G	C	T	T	G	A	T	G	A	C

Accession number	1017	1019	1023	1026	1029	1032	1034	1061	1063	1064	1065	1067	1068	1071	1076
NM_001364810.1 l *Cicer arietinum*	A	A	A	C	A	A	G	A	A	A	G	T	A	A	A
XM_003592050.3 l *Medicago truncatula*	A	A	A	C	A	A	G	A	A	A	G	T	A	A	A
KF658495.1 l *Vicia faba*	A	A	A	C	A	A	G	A	A	A	G	T	A	A	A
XM_025819576.1 l *Arachis hypogaea*	A	A	A	C	A	A	G	A	A	A	G	T	A	A	A
XM_021131527.1 l *Arachis duranensis*	A	A	A	C	A	A	G	A	A	A	G	T	A	A	A
Cajanus cajan POU domain	A	A	A	C	A	A	G	A	A	A	G	T	A	A	A
XM_025765336.1 l *Arachis hypogaea*	A	A	A	C	A	A	G	A	A	A	G	T	A	A	A
JX082400.1 l *Phaseolus vulgaris*	A	A	A	C	A	A	G	A	A	A	G	T	A	A	A
JX082401.1 l *Phaseolus vulgaris*	A	A	A	C	A	A	G	A	A	A	G	T	A	A	A
XM_028366588.1 l *Glycine soja*	A	A	A	C	A	A	G	A	A	A	G	T	A	A	A
NM_001250558.2 l *Glycine max*	A	A	A	C	A	A	G	A	A	A	G	T	A	A	A
XM_028078512.1 l *Vigna unguiculata*	A	A	A	C	A	A	G	A	A	A	G	T	A	A	A

Accession number	1078	1079	1082	1085	1097	1098	1100	1102	1103	1106	1107	1108	1109	1110	1118
NM_001364810.1 l *Cicer arietinum*	G	T	C	C	C	T	C	G	T	G	A	T	C	T	T
XM_003592050.3 l *Medicago truncatula*	G	T	C	C	C	T	C	G	T	G	A	T	C	T	T
KF658495.1 l *Vicia faba*	G	T	C	C	C	T	C	G	T	G	A	T	C	T	T
XM_025819576.1 l *Arachis hypogaea*	G	T	C	C	C	T	C	G	T	G	A	T	C	T	T
XM_021131527.1 l *Arachis duranensis*	G	T	C	C	C	T	C	G	T	G	A	T	C	T	T
Cajanus cajan POU domain	G	T	C	C	C	T	C	G	T	G	A	T	C	T	T
XM_025765336.1 l *Arachis hypogaea*	G	T	C	C	C	T	C	G	T	G	A	T	C	T	T
JX082400.1 l *Phaseolus vulgaris*	G	T	C	C	C	T	C	G	T	G	A	T	C	T	T
JX082401.1 l *Phaseolus vulgaris*	G	T	C	C	C	T	C	G	T	G	A	T	C	T	T
XM_028366588.1 l *Glycine soja*	G	T	C	C	C	T	C	G	T	G	A	T	C	T	T
NM_001250558.2 l *Glycine max*	G	T	C	C	C	T	C	G	T	G	A	T	C	T	T
XM_028078512.1 l *Vigna unguiculata*	G	T	C	C	C	T	C	G	T	G	A	T	C	T	T

Accession number	1120	1183	1184	1185	1191	1204	1205	1206	1207	1208
NM_001364810.1 l *Cicer arietinum*	G	C	T	T	A	C	A	T	C	A
XM_003592050.3 l *Medicago truncatula*	G	C	T	T	A	C	A	T	C	A
KF658495.1 l *Vicia faba*	G	C	T	T	A	C	A	T	C	A
XM_025819576.1 l *Arachis hypogaea*	G	C	T	T	A	C	A	T	C	A
XM_021131527.1 l *Arachis duranensis*	G	C	T	T	A	C	A	T	C	A
Cajanus cajan POU domain	G	C	T	T	A	C	A	T	C	A
XM_025765336.1 l *Arachis hypogaea*	G	C	T	T	A	C	A	T	C	A
JX082400.1 l *Phaseolus vulgaris*	G	C	T	T	A	C	A	T	C	A
JX082401.1 l *Phaseolus vulgaris*	G	C	T	T	A	C	A	T	C	A
XM_028366588.1 l *Glycine soja*	G	C	T	T	A	C	A	T	C	A
NM_001250558.2 l *Glycine max*	G	C	T	T	A	C	A	T	C	A
XM_028078512.1 l *Vigna unguiculata*	G	C	T	T	A	C	A	T	C	A
