# Supplementary material for: Characterization of ASR gene and its role in drought tolerance in chickpea (Cicer arietinum L.)
Source: PLoS One. 2020 Jul 14;15(7):e0234550. doi: 10.1371/journal.pone.0234550 (PMC7360048; doi:10.1371/journal.pone.0234550)
Supplement: S1 Raw image — PCR amplification of seven chickpea genotypes viz., ICCV97309, ICCV3311, ICCV10316, ICCV9307, BGD72, ICCV10, and ICCV5313 was done using ASR gene specific primer and revealed a single amplicon ranging from 680-700bp; Marker-100 bp Banglore Genei DNA ladder. (PDF) [file pone.0234550.s002.pdf]

S2 file

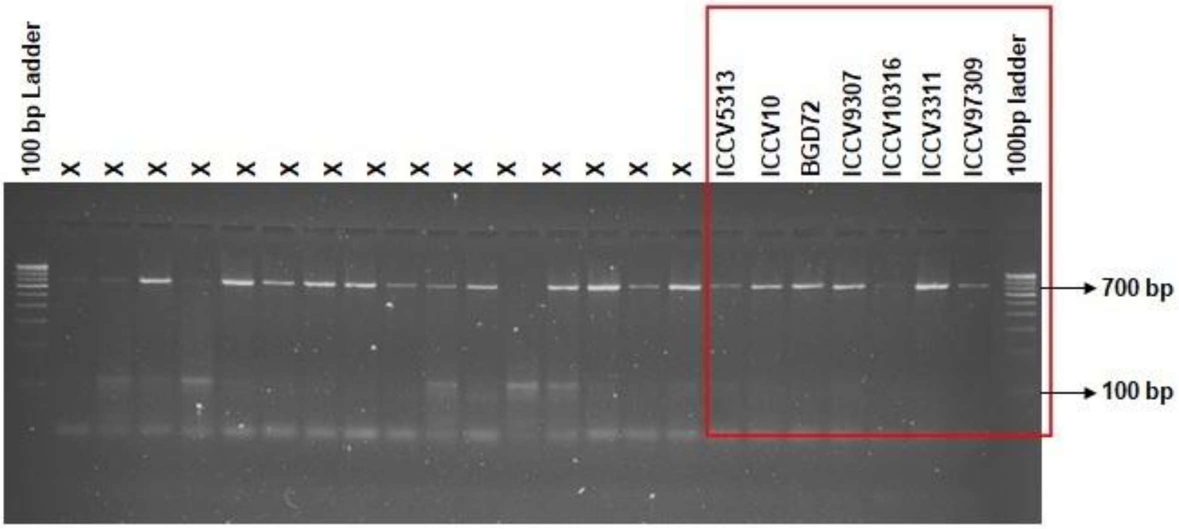

S3 Raw image. PCR Amplification of seven chickpea genotypes using ASR gene specific marker. PCR amplification of seven chickpea genotypes *viz*., ICCV97309, ICCV3311, ICCV10316, ICCV9307, BGD72, ICCV10, and ICCV5313 was done using ASR gene specific primer and revealed a single amplicon ranging from 680-700bp; Marker-100 bp Banglore Genei DNA ladder.
